# Lifting the veil on tumor metabolism: A GDH1-focused perspective

**DOI:** 10.1016/j.isci.2025.112551

**Published:** 2025-05-03

**Authors:** Sisi Zhou, Huaer Wu, Yun Chen, Jiawei Lv, Shufang Chen, Hua Yu, Tiezhu Shi, Xiongjun Wang, Lingyun Xiao

**Affiliations:** 1 Precise Genome Engineering Center, School of Life Sciences, Guangzhou University, Guangzhou 510006, China

**Keywords:** Cancer

## Abstract

Tumors depend on glutamine for energy production, biosynthesis, and redox homeostasis. Glutamate dehydrogenase 1 (GDH1) primarily catalyzes the oxidative deamination of glutamate to α-ketoglutarate (α-KG) and ammonia, utilizing NAD^+^ or NADP^+^ as cofactors. α-KG is a tricarboxylic acid (TCA) cycle intermediate at the nexus of multiple metabolic pathways, fueling the TCA cycle for energy production or providing intermediates essential for lipid, amino acid, and nucleotide synthesis. Its derivatives, succinate and fumarate, function as oncometabolites that promote tumor progression through diverse mechanisms. Additionally, α-KG is an essential cofactor for α-KG-dependent dioxygenases (2-OGDDs), regulating epigenetic modifications that drive tumorigenesis. GDH1 may also catalyze the reductive amination of α-KG to glutamate under glutamine deprivation or hypoxic conditions. The roles of GDH1 in tumors are context-dependent, influencing progression through metabolic and epigenetic mechanisms. This review discusses GDH1’s multifaceted functions and advances in targeting it for cancer therapy.

## Graphical abstract

**Figure:**
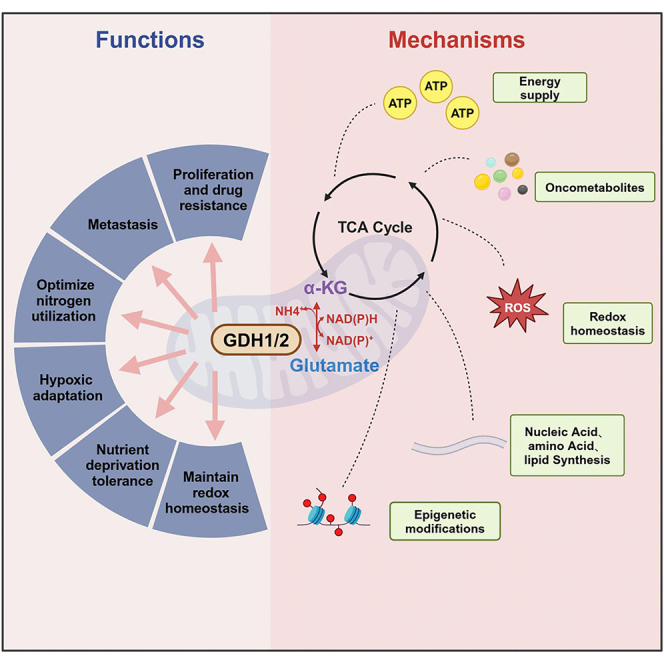

## Introduction

Over the past few decades, metabolic reprogramming has been recognized as a hallmark of cancer.^1^^,^^2^ Increased aerobic glycolysis is the most well-known altered metabolic pathway. As a consequence, there is a reduction in mitochondrial ATP production, while the need for biosynthetic precursors and NADPH is on the rise.^3^^,^^4^ To counterbalance these changes, tumor cells frequently depend on glutamine for proliferation and metastasis, a phenomenon termed “glutamine addiction”, wherein glutamine serves as a key substrate for energy production, biosynthesis, and redox homeostasis.^5^^,^^6^^,^^7^^,^^8^^,^^9^

**Figure 1:**
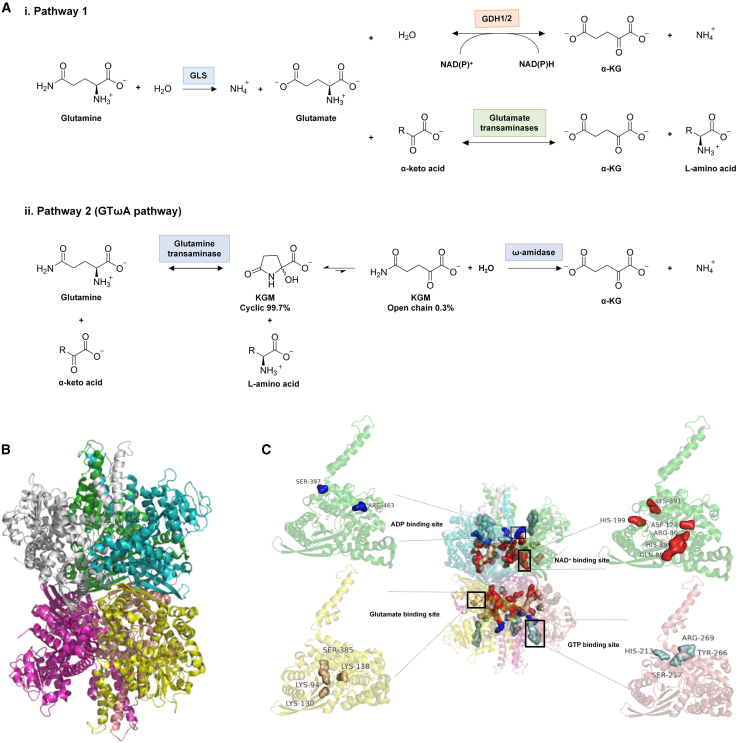
Enzymatic reaction and structure of GDH1 (A) Two distinct pathways are involved in glutamine utilization to generate α-KG. The first pathway (pathway 1; the canonical pathway) involves the hydrolysis of glutamine by GLS to glutamate, followed by its conversion to α-KG catalyzed by GDH1/2 or glutamate transaminases, such as GOT, GPT, and PSAT. The second pathway is the GTωA pathway, where glutamine is transaminated to KGM by glutamine transaminase, which is subsequently hydrolyzed to α-KG by ω-amidase. (B) The crystal structure of human GDH1 as a hexamer (PDB: 1L1F). Each subunit is shown in different colors. (C) The binding sites of glutamate (yellow), ADP (blue), GTP (palecyan), and NAD^+^ (Red) in human GDH1. These substrates and allosteric regulators bind to distinct sites. Abbreviation: α-KG, α-ketoglutarate; GDH1/2, glutamate dehydrogenase 1; GLS, glutaminase; GOT, glutamate oxaloacetate transaminase; GPT, glutamate pyruvate transaminase; KGM, α-ketoglutaramate; PSAT, phosphoserine aminotransferase.

Two distinct pathways are involved in glutamine utilization to generate α-ketoglutarate (α-KG, also known as 2-oxoglutarate, 2-OG), a crucial mediator in glutamine addiction (Figure 1A).^6^^,^^10^^,^^11^^,^^12^^,^^13^ The first pathway (pathway 1; the canonical pathway) involves the hydrolysis of glutamine by glutaminase (GLS) to glutamate, followed by its conversion to α-KG catalyzed by glutamate dehydrogenase (GDH1/2, also known as GLUD1/2) or glutamate transaminases, such as aspartate aminotransferase (AST, also known as glutamate oxaloacetate transaminase, GOT), alanine aminotransferase (ALT, also known as glutamate pyruvate transaminase, GPT), and phosphoserine aminotransferase (PSAT).^13^^,^^14^ The second, less explored pathway involves glutamine transaminase and ω-amidase (GTωA pathway; glutaminase II pathway; pathway 2), where glutamine is transaminated to α-ketoglutaramate (KGM) by glutamine transaminase, which is subsequently hydrolyzed to α-KG by ω-amidase.^10^^,^^15^^,^^16^ A key benefit of the GTωA pathway is its irreversible nature and ability to produce α-KG during hypoxia without altering the redox balance. However, research of this pathway on tumors is currently limited, and its role has been summarized in another study.^10^ These enzymes might play nonredundant roles in different tumors.

Recent studies have progressively elucidated the pivotal roles of GDH1/2 in glutamine addiction in cancer cells.^3^^,^^13^^,^^17^^,^^18^^,^^19^ Human GDH consists of two isoenzymes, GDH1 and GDH2, which catalyze the same biochemical reaction, though they differ in regulation and tissue distribution. GDH1 is widely expressed across various tissues, while GDH2 is selectively expressed in the retina, brain, kidney, and steroidogenic organs. Notably, GDH1 is present in most tumors, whereas GDH2 is detectable in only a limited subset, resulting in significantly more research on GDH1 in tumor biology.^20^^,^^21^^,^^22^ GDH1 catalyzes the reversible conversion of glutamate to α-KG and ammonia in the presence of NAD^+^ or NADP^+^ as cofactors.^18^^,^^23^^,^^24^ Although reversible, GDH1 predominantly facilitates the oxidative deamination of glutamate to α-KG,^18^^,^^25^^,^^26^^,^^27^ which is a pivotal intermediate in the tricarboxylic acid (TCA) cycle, standing at the crossroads of numerous metabolic pathways.^3^^,^^28^^,^^29^^,^^30^^,^^31^ α-KG can fuel the TCA cycle to generate ATP, particularly under conditions where glycolysis is impaired, thereby sustaining cell survival. α-KG also supports the reprogramming of the TCA cycle by replenishing intermediates essential for biosynthesis. For example, citrate, generated through the reductive carboxylation of α-KG, is converted to acetyl-CoA in the cytosol by ATP citrate lyase (ACLY), initiating lipid biosynthesis.^29^^,^^32^ α-KG-derived oncometabolites in the TCA cycle, such as succinate and fumarate, promote tumor progression through multiple mechanisms.^30^^,^^33^^,^^34^^,^^35^^,^^36^ Both fumarate and succinate stabilize hypoxia-inducible factor 1α (HIF-1α) by inhibiting prolyl-4-hydroxylase domain-containing proteins (PHDs), thereby inducing a pseudohypoxic state that fosters inflammation, angiogenesis, and metastasis.^30^^,^^37^ These metabolites also influence redox homeostasis and suppress host anti-tumor defenses.^3^^,^^33^^,^^36^ For instance, fumarate directly activates glutathione peroxidase 1 (GPx1), enhancing redox balance and increasing chemotherapy resistance.^3^ Beyond its metabolic roles, α-KG is an obligatory co-substrate for α-KG-dependent dioxygenases (αKGDs, also known as 2-oxoglutarate-dependent dioxygenases, 2-OGDDs), a diverse group of enzymes that catalyze C-H bond oxidation and hydroxylation of various substrates.^30^^,^^38^ It is estimated that humans possess 60–70 2-OGDDs, which include PHDs, factor inhibiting FIH, Jumonji C domain-containing histone lysine demethylases (JmjC-KDMs), ten-eleven translocation (TET) family proteins, ALKB homolog (ALKBH) proteins, and lysyl hydroxylases (LHs).^30^^,^^38^^,^^39^^,^^40^^,^^41^ Consequently, α-KG has garnered increasing attention for its expanding roles in epigenetic regulation and stress response. NADH, generated by GDH1, acts as the reducing equivalent for ATP production through oxidative phosphorylation (OXPHOS).^42^ In addition, GDH1-derived NADPH provides reducing power for anabolic reactions and supports redox homeostasis.^20^^,^^21^^,^^43^ Under specific conditions, GDH1 may shift its reaction direction toward reductive amination to mediate ammonia fixation.^44^^,^^45^^,^^46^^,^^47^^,^^48^^,^^49^ For instance, GDH1 is involved in ammonia recycling, maximizing nitrogen utilization to support tumor growth.^47^

The glutamine metabolic pathway varies across different tumors, with each tumor exposed to distinct metabolite concentrations. GDH1 is upregulated in various tumors, where it is associated with altered tumor proliferation, migration, and resistance to adverse environmental stress.^41^^,^^50^^,^^51^^,^^52^ For instance, GDH1 contributes to anaplerosis of the TCA cycle, particularly under glucose-deprived conditions. Emerging mechanisms by which GDH1 promotes tumor progression have been elucidated in specific tumor types, including the upregulation of glucose transport,^19^ stabilization of HIF-1α,^45^ regulation of redox homeostasis,^3^^,^^53^ induction of EMT and anoikis,^54^^,^^55^^,^^56^ and mediation of ammonia recycling.^47^ Understanding the complexities of altered glutamine metabolism and the role of GDH1 in tumors could aid the development of innovative cancer therapies. This paper aims to provide an overview of the multifaceted role of GDH1 in tumors and recent advances in GDH1 inhibitors, focusing on cancer therapy to inspire the development of GDH1-based cancer therapeutics.

## Genomics of human *GLUD*

Humans express two GDH isoenzymes (GDH1 and GDH2) with different genetic origins.^26^^,^^27^^,^^57^^,^^58^ GDH1 is encoded by the *GLUD1* gene, which is organized into 13 exons and is located on human chromosome 10. *GLUD1* is recognized as a widely expressed housekeeping gene, expressed in nearly all tissues, with its highest levels found in the liver.^59^^,^^60^^,^^61^ The *GLUD2* gene, encoding GDH2, is located on the X chromosome and selectively expressed in the retina, brain, kidney, and steroidogenic organs such as adrenal, ovary, placenta, and testis.^58^^,^^61^^,^^62^ *GLUD2* was thought to emerge in hominoids through retroduplication after the Old World monkey-hominoid split, but before the separation of the gibbon lineage from that of humans and great apes, around 18–23 million years ago.^63^ The amino acid substitutions that occurred before the separation of the gibbon from the human lineage were E34K, D142E, S174N, R443S, G456A, and N498S, whereas the substitutions were S331T, M370L, and R470H before the separation of humans from orangutans.^63^^,^^64^ These changes in GDH2 were thought to be under positive natural selection. In contrast, *GLUD1* is highly conserved through strong purifying selection.

## Subcellular location of GDH

The subcellular localization of GDH1 and GDH2 provides insights into their biological roles. Both isoenzymes are primarily localized in the mitochondria, as confirmed by green fluorescent protein (GFP) tagging.^59^^,^^65^^,^^66^^,^^67^ Co-expression in 293T and HeLa cells further demonstrated their colocalization in mitochondrial structures.^59^ A small fraction of GDH1 and GDH2 is also associated with the endoplasmic reticulum in an unprocessed form, with the leader sequence intact.^65^ Rosso et al. reported that GDH1 may also localize to the cytoplasm, whereas GDH2 is specifically targeted to the mitochondria.^67^ The enhanced mitochondrial targeting of hGDH2, compared to hGDH1, is attributed to the E7K and D25H substitutions in the long cleavable presequence.

In the human brain, immunostaining showed that GDH1 was expressed in glial cells (astrocytes, oligodendrocytes, and oligodendrocyte precursors), whereas GDH2 was found in both astrocytes and neurons.^68^ In astrocytes, GDH1 was primarily mitochondrial but also associated with the nuclear membrane. In oligodendrocytes and oligodendrocyte precursors, GDH1 localized predominantly to the nucleus. GDH2, in contrast, was localized to the nuclear membrane in small neurons with dark nuclei, while in larger cortical neurons it was distributed throughout the cytoplasm in structures resembling mitochondria.

## Sequence alignment of GDH isoenzymes

Alternative splicing of the *GLUD* genes results in the production of four isoforms with distinct molecular masses and isoelectric points in humans.^69^ *GLUD1* generates three GDH1 isoforms (a, b, and c) of 558, 425, and 391 amino acids, respectively, with isoform a being the predominant form and the most widely studied in the literature.^26^^,^^27^^,^^57^^,^^59^^,^^70^ These GDH1 isoforms arise from differences in their first and second exons.^57^ In contrast, *GLUD2* produces a single GDH2 isoform of 558 amino acids.

GDH1 (isoform a, as referred to in publications) and GDH2 both contain the first 53 amino acids at the N-terminus, forming the leader peptide that facilitates their transport into the mitochondrial matrix.^57^^,^^71^^,^^72^ After cleavage of the leader peptide, the mature GDH1 and GDH2 isoenzymes are highly homologous, differing in only 15 of their 505 amino acid residues. Interestingly, the proportion of substitutions in the mitochondrial targeting signal (9/53) is significantly higher than that in the rest of the sequence (15/505).^57^

## GDH1-mediated enzymatic reaction

GDH1 catalyzes the reversible conversion of glutamate to α-KG using NAD(P)^+^ as a coenzyme. There has been debate on the reaction directionality of GDH1 toward the formation or breakdown of glutamate, which is determined by the concentration of substrates and the enzyme’s affinity (*K*_M_ value) for these substrates.^27^ After reviewing the *K*_M_ values for GDH1 substrates at different pH levels, along with their physiological concentrations, it was speculated that in most cases GDH1 primarily mediates the oxidative deamination of glutamate.^18^^,^^25^^,^^26^^,^^73^^,^^74^^,^^75^^,^^76^ NAD^+^ is the primary coenzyme utilized by GDH1, and the *in vivo* NAD^+^ level (0.4–1.7 mM) significantly surpasses the *in vitro K*_M_ value (0.01–0.2 mM).^26^^,^^77^ The *K*_M_ for ammonium (13–164 mM) is exceptionally high, hundreds of times higher than the typical serum ammonium level (less than 50 μM), making glutamate synthesis via GDH1 *in vivo* improbable.^25^^,^^26^^,^^73^ Several tracer studies in insulinoma cells and neurons have confirmed that GDH1 functions in the oxidative deamination direction.^78^^,^^79^^,^^80^

Notably, GDH1 catalysis is affected by pH, with high pH promoting reductive amination and low pH favoring oxidative deamination.^18^^,^^73^^,^^81^ The concentration of metabolites exhibits significant variability both between tissues and within them, and local ammonia levels are higher inside mitochondria compared to other compartments, making the enzymatic direction of GDH1 not absolute.^47^^,^^73^^,^^82^ GDH1 in the liver is expected to catalyze reactions in both directions, given that hepatocytes encounter fluctuating levels of glutamate and ammonia levels, particularly after a high-protein meal.^83^^,^^84^^,^^85^^,^^86^^,^^87^ In the brain, the elevated glutamate levels typically promote the oxidative deamination activity of GDH1, which is coordinated with the swift detoxification of ammonia by glutamine synthetase (GS).^78^^,^^88^ However, under hyperammonemic conditions, ammonia in the brain can reach millimolar levels, and GDH1 has been reported to be important for ammonia fixation.^48^ Similarly, GDH2 contributes to ammonia clearance through the reductive amination of α-KG.^44^^,^^49^ In tumors, ammonia accumulates due to limited blood supply and increased glutamine catabolism, prompting GDH1 to participate in ammonia recycling to support cellular growth.^47^

## Allosteric regulation of GDH1

Under most conditions, the rate-limiting step for GDH1 is coenzyme release.^89^ Abortive complexes are readily formed, particularly at higher substrate concentrations, when the product is replaced by the substrate and the reacted coenzyme has not dissociated.^57^^,^^73^^,^^90^^,^^91^^,^^92^ For example, GDH1·glutamate·NAD(P)H is the abortive complex in the oxidative deamination reaction, while GDH1·α-KG·NAD(P)^+^ serves as the abortive complex in the reductive amination reaction. The tight binding of the coenzyme to GDH1 significantly slows down enzymatic turnover, with an initial burst phase in pre-steady-state kinetics.^90^^,^^93^^,^^94^ The stability of abortive complexes can modulate the GDH1-catalyzed reaction.

GDH1 is subject to extensive allosteric regulation, where its activity is modulated by effectors binding to sites distinct from the active site.^23^^,^^95^ Inhibitors of GDH1 include GTP,^96^^,^^97^ ATP,^98^ NADH,^97^ and palmitoyl CoA,^99^ while ADP,^97^ GDP,^100^^,^^101^ and leucine^102^^,^^103^ serve as allosteric activators. Among these, GTP and ADP are the primary endogenous negative and positive modulators, respectively.^24^^,^^25^ GTP inhibits GDH1 when energy is abundant, and reduced GTP along with increased ADP levels activate GDH1 to enhance α-KG flux under conditions of limited acetyl-CoA, supporting the TCA cycle.^23^ NADH binding enhances subsequent GTP binding, and vice versa*.*^96^ Leucine is a poor substrate for GDH1 but serves as an allosteric activator akin to ADP.^103^^,^^104^ NADH competes with ADP for the same binding site, but they exert distinct effects on the enzyme.^26^^,^^96^ These metabolites regulate GDH1 activity both agonistically and antagonistically, enabling the enzyme to adapt dynamically to varying cellular conditions.

It was reported that these allosteric regulators likely act by stabilizing or destabilizing the abortive complex of GDH1.^26^^,^^90^^,^^91^^,^^92^^,^^104^ For instance, GTP binds to the base of the antenna, wedged between the NAD binding domain and the pivot helix, with this binding site accessible only when the catalytic cleft is closed.^26^^,^^105^ Therefore, GTP inhibits the reaction by slowing down product release via stabilizing the GDH1·glutamate·NAD(P)H complex.^26^^,^^97^ ATP binds to the same site as GTP on GDH1, inhibiting GDH1 activity at concentrations below 100 μM, with an ED_50_ (>10 μM) much higher than that of GTP (42 nM).^26^^,^^106^ In contrast, ADP decreases product affinity and facilitates the dissociation of abortive complexes, thereby activating GDH1.^90^^,^^107^

## Structure of GDH1

The structure of human GDH1 has been extensively characterized.^18^^,^^25^^,^^71^^,^^90^^,^^108^ It adopts a symmetric hexameric configuration, consisting of six identical subunits arranged as a dimer of trimers (Figure 1B). Each subunit contains the N-terminal glutamate binding domain, NAD(P) binding domain, antenna, pivot helix, and C-terminal helices (Figure 1C). The two trimeric N-terminal glutamate-binding domains form the enzyme’s core. The NAD(P) binding domain, located above the glutamate-binding domain, contains the conserved nucleotide-binding motif. *K*_M_ values for glutamate were 4- to 10-fold higher in mutants at the K94, G96, or K118 sites compared to wild-type GDH1 and GDH2, while the K130 mutation mainly affected the *k*_cat_ value, suggesting a possible involvement of K130 in catalysis.^109^ These mutants did not alter the allosteric regulation by ADP or GTP, however, D172Y showed reduced sensitivity to ADP activation.^109^ C323 mutation in NAD(P) binding domain of GDH1 and GDH2 resulted in a significant decreased catalytic efficiency (*k*_cat_/*K*_M_).^64^^,^^110^ The pivot helix is part of the NAD(P) binding domain, and contributes to catalysis through its rotating regions. The antenna, a 50-amino acid α-helical structure extending above the NAD binding domain, intertwines with the antennas of adjacent subunits. The pivot helix and antenna structure form a characteristic regulatory domain in human GDH1.^18^^,^^90^

Numerous studies have emphasized the pivotal role of the pivot helix and antenna structure in regulating the basal activity, allosteric modulation, and thermal stability of human GDH1.^26^^,^^70^^,^^106^^,^^111^^,^^112^ The 50-residue antenna is specific to mammalian GDH.^57^^,^^90^^,^^104^ A truncated version of the antenna is present in ciliate GDH, which shows allosteric activation by ADP and inhibition by palmitoyl-CoA.^23^ Replacing the antenna of human GDH1 (residues 398–448) with the short loop from *Clostridium symbiosum* GDH abolishes sensitivity to regulation by ADP, GTP, and palmitoyl-CoA.^113^ Another study demonstrated that an antenna-less GDH1 mutant (deleting residues 402–442, without the bacterial loop) exhibited a significant reduction in basal activity, retaining only 13% of the wild-type activity, but showed a ∼100-fold increase in sensitivity to all allosteric activators, while GTP inhibition was reduced.^90^ The R400Q mutation in the antenna-less mutant abolished ADP activation without affecting GTP inhibition. The R463A mutation in the pivot helix impaired ADP activation with minimal impact on GTP inhibition or leucine activation.^90^^,^^114^

Hyperinsulinism/hyperammonemia (HI/HA) syndrome is characterized by fasting- and protein-sensitive hypoglycemia, along with persistently elevated serum ammonia levels.^115^ HI/HA is linked to mutations in GDH1 that increase enzyme activity and impair GTP inhibition. Several GDH1 mutations involved in HI/HA, including N410, L413, F440, Q441, R443, S445, G446, A447, and S448, are localized to the descending helix of the antenna.^106^^,^^108^^,^^115^^,^^116^^,^^117^ For example, the R443W, S445L, and G446C mutations increase the GTP IC_50_ by 600- to 800-fold.^90^ Two mutations in the pivot helix (K450 and H454) alter amino acids that directly bind GTP, leading to GTP inhibition resistance while preserving basal catalytic activity.^70^^,^^118^ Mutations in the GTP-binding site (S217, R221, H262, R265, Y266, and R269) abolish the interaction between mutant GDH1 and GTP, thereby eliminating the inhibitory effect of GTP.^115^

It has been reported that the trimeric antenna in GDH1 undergoes significant conformational changes during catalysis, which are crucial for allosteric regulation.^27^^,^^71^^,^^90^^,^^108^^,^^119^ The helix appears to hyperextend upon the opening of the active site and recoils during closure.^90^ Consequently, the regulatory domain lowers the energy required to open and close the catalytic cleft. Removing the antenna may increase this energy or stabilize a “GTP-inhibited-like” state, thereby reducing enzyme turnover rate. Mutations in the regulatory domains can stabilize the helix, which is essential for allosteric function.^90^^,^^119^ Additionally, mutations may create an unfavorable charged environment for GTP binding.^71^

## Evolutionary mutations from GDH1 to GDH2

The comparison between human GDH1 and GDH2 also provides evidence for allosteric regulation in the pivot helix and antenna. The mature GDH2 is highly homologous to GDH1 but possesses distinct features, including lower basal activity, lower optimum pH, enhanced activation by ADP and leucine, increased GTP resistance, and reduced heat stability.^25^^,^^64^^,^^71^ A key evolutionary change, R443S in the antenna of GDH2, results in a baseline activity of less than 10% of its full capacity upon full activation by ADP/leucine.^112^ In contrast, GDH1 displays 35–40% basal activity without allosteric effectors. Additionally, R443S in GDH2 alters sensitivity to ADP and leucine without affecting GTP inhibition, while rendering the enzyme extremely heat labile.^112^^,^^120^^,^^121^ Another evolutionary change, the G456A mutation in the pivot helix, confers GTP resistance in GDH2.^111^ However, the double mutant of R443S and G456A was GTP sensitive, whereas the triple mutants of M415L, R443S, and G456A acquired GTP inhibition resistance properties.^122^ The S445 mutation in GDH2 confers resistance to GTP regulation while enhancing sensitivity to estrogen inhibition, resulting in accelerated Parkinson’s disease onset in hemizygous males but not in heterozygous females.^123^ Kanavouras et al. demonstrated that mutations in the antenna (Q441R and S445L), pivot helix (K450E, H454Y), and the junction between these regions (S448P) differentially affect basal activity, allosteric regulation, and thermal stability of GDH2.^124^

Co-expression of GDH1 and GDH2 in several tissues may confer a biological advantage.^18^^,^^25^ These enzymes are expressed in astrocytes, which are involved in the removal and metabolism of transmitter glutamate, supplying neurons with glutamine and lactate.^18^ In the renal tubule epithelium, their presence is vital for energy-demanding renal processes.^25^^,^^125^ Additionally, GDH1/2 generate NADPH in mitochondria, supporting steroid hormone biosynthesis in steroidogenic cells.^61^

Due to its lower optimal pH, GDH2 may function more efficiently than GDH1 under conditions of acidification.^25^ For instance, GDH2 expressed in the epithelial cells of the proximal convoluted tubules could facilitate the excretion of excess protons as ammonium during acidosis.^25^ GDH2 may also have adapted to the acidic environment of nerve tissue during excitatory transmission, particularly in synaptic astrocytes following glutamate uptake.^64^^,^^126^ Notably, GDH2, rather than GDH1, may play a critical role in promoting tumor development in tumors with the R132H mutation in isocitrate dehydrogenase 1 (IDH1).^127^^,^^128^ IDH1^R132H^ generates high concentrations of D-2-hydroxyglutarate (D-2-HG) from α-KG, with GDH2 aligning with the acidified intracellular conditions induced by D-2-HG, while GDH1 activity is significantly inhibited. Sertoli cells specifically express GDH2, which supports germ cells by providing lactate via the glutamate-TCA cycle.^18^^,^^27^ Germ cells preferentially use lactate over glucose to maintain high ATP levels for sperm motility. Disruption of Sertoli cell function reduces lactate production, leading to spermatogenic failure. The high expression of GDH2 suggests that glutamate metabolism complements glycolysis as an alternative pathway for lactate production.

The relatively low basal activity and enhanced activation of GDH2 (by 2400% at 1.0 mM ADP) enable it to support robust glutamate metabolism during bursts of glutamatergic firing required for long-term potentiation.^129^ It has been shown that GDH2 potentiates glutamatergic transmission in the hippocampus, thereby enhancing synaptic plasticity.^25^^,^^129^^,^^130^

## The role of GDH1 in tumor biology

### GDH1 generates α-KG to replenish the TCA cycle

**Figure 2:**
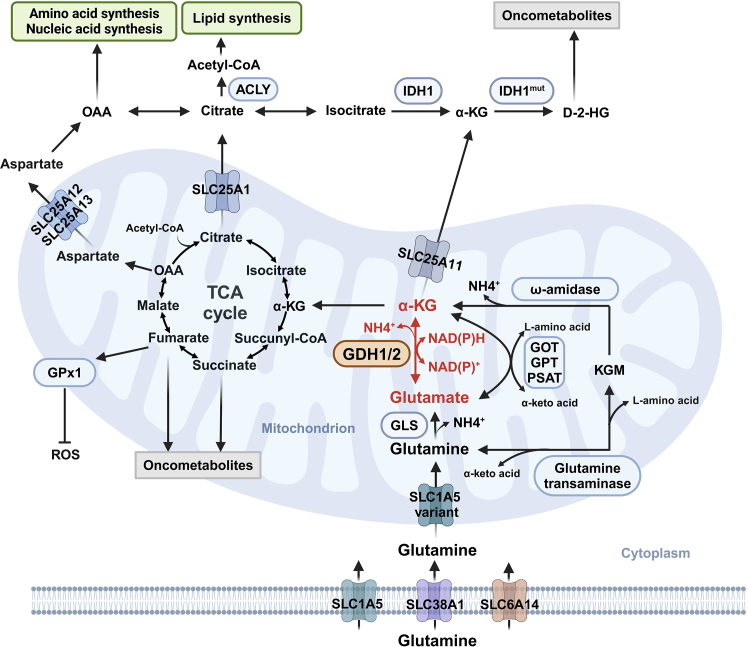
Metabolic and oncogenic roles of GDH1 GDH1/2 catalyzes the reversible conversion of glutamate to α-KG and ammonia, using NAD^+^ or NADP^+^ as cofactors. Glutamate transaminases, including GOT, GPT, and PSAT, mediate the reversible transfer of amino groups between glutamate and α-keto acids, generating α-KG and L-amino acids. The GTωA pathway produces α-KG from glutamine via a distinct route. These enzymes play nonredundant roles in different tumors. GDH1 primarily facilitates the oxidative deamination of glutamate to α-KG, a key TCA cycle intermediate that links energy metabolism and biosynthesis. α-KG fuels ATP production or replenishes TCA cycle intermediates, such as citrate for lipid synthesis. α-KG-derived succinate and fumarate act as oncometabolites that promote tumor progression through multiple mechanisms. In IDH1-mutant tumors, such as those with IDH1^R132H^, α-KG is converted to the oncometabolite D-2-HG, leading to intracellular acidification, which favors GDH2 activity but is less compatible with GDH1. Under specific conditions, GDH1 can mediate ammonia fixation via reductive amination. Abbreviation: α-KG, α-ketoglutarate; ACLY, ATP citrate lyase; D-2-HG, D-2-hydroxyglutarate; GDH1/2, glutamate dehydrogenase 1/2; GLS, glutaminase; GOT, glutamate oxaloacetate transaminase; GPx1, glutathione peroxidase 1; GPT, glutamate pyruvate transaminase; IDH1, Isocitrate dehydrogenase 1; KGM, α-ketoglutaramate; OAA, oxaloacetate; PSAT, phosphoserine aminotransferase; ROS, reactive oxygen species. Created in BioRender. Sisi, Z. (2025) https://BioRender.com/k39e278.

The ‘glutamate family' of amino acids consists of arginine, ornithine, proline, histidine, and glutamine, where their conversion into glutamate is a key step in their metabolic disposal.^131^^,^^132^ Mitochondrial GDH1 further metabolizes glutamate to produce α-KG, a pivotal intermediate in the TCA cycle (Figure 2).^3^^,^^28^^,^^29^^,^^30^^,^^31^ α-KG fuels both energetic and anabolic pathways. It may be oxidized by the α-KG dehydrogenase complex (α-KGDC) to produce succinyl-CoA, or reduced, pushing the TCA cycle toward citrate. Additionally, α-KG enters the TCA cycle to generate ATP or modulate the levels of multiple TCA intermediates.^29^^,^^34^ These intermediates can be transported into the cytosol, where they function as oncometabolites or contribute to macromolecule synthesis. For example, retinal pigment epithelium cells exhibit a high capacity for reductive carboxylation via NADPH-dependent IDH, producing excess citrate, which is then used for lipid synthesis.^133^ In contrast, IDH1 mutations, such as IDH1^R132H^, consume α-KG and NADPH, leading to the production of D-2-HG.^127^^,^^128^ Mutations in succinate dehydrogenase (SDH) and fumarate hydratase (FH) result in elevated succinate and fumarate levels.^134^^,^^135^^,^^136^ 2-HG, succinate, and fumarate serve as structural mimics of α-KG and competitively inhibit a superfamily of 2-OGDDs.^34^^,^^35^^,^^36^^,^^137^^,^^138^ These are well-established oncometabolites that induce distinctive biological patterns in tumors, including hypermethylation phenotypes, metabolic reprogramming, and altered redox homeostasis.

Glutamine is the most abundant amino acid and serves as a major carbon source for the TCA cycle.^139^^,^^140^ Two distinct pathways contribute to glutamine-derived α-KG production: the canonical pathway, mediated by GLS and subsequently by GDH1/2 or glutamate transaminases, and the GTωA pathway (Figure 1A).^6^^,^^10^^,^^11^^,^^12^^,^^13^ These enzymes may play nonredundant roles in different tumors, but their functions require further comprehensive investigation. Due to its widespread distribution, GDH1 is considered crucial in replenishing TCA cycle intermediates, thereby influencing cancer development.^31^^,^^141^^,^^142^^,^^143^

### GDH1 produces α-KG to regulate 2-OGDDs

2-OGDDs are a diverse group of enzymes that catalyze C-H bond oxidation and hydroxylation of various substrates.^30^^,^^38^ The 2-OGDDs superfamily includes PHDs, FIH, JmjC-KDMs, TET, ALKBH, and LH.^30^^,^^38^^,^^39^^,^^40^^,^^41^ α-KG, an obligatory co-substrate, emerges as a key factor governing the activities of 2-OGDDs, which rely on additional O_2_ and Fe^2+^ to carry out their enzymatic functions. In contrast, succinate, fumarate, and 2-HG compete with α-KG, thereby inhibiting the activity of 2-OGDDs.^34^^,^^35^^,^^36^^,^^137^^,^^138^

As 2-OGDDs require O_2_ for their enzymatic activities, PHDs and FIH function as key oxygen sensors within cells.^38^^,^^144^^,^^145^^,^^146^^,^^147^^,^^148^ Under normal conditions, PHDs hydroxylate specific proline residues on HIF-1α and HIF-2α, promoting their proteasomal degradation. FIH hydroxylates an asparagine residue on HIF-1α and HIF-2α, inhibiting their interaction with co-activators like p300/CREB-binding protein (CBP) and modulating their transcriptional activities.^38^ Conversely, under hypoxic conditions (0.25–1% O_2_), PHD activity declines, whereas FIH activity remains unchanged until oxygen concentration approaches near anoxic levels (0.1% O_2_). In cancer cells harboring mutant IDH1, SDH, or FH, excess oncometabolites (such as D-2-HG, succinate, or fumarate) accumulate, inhibiting PHD and FIH activity, thereby driving cancer progression and therapy resistance.^38^^,^^39^^,^^149^

**Figure 3:**
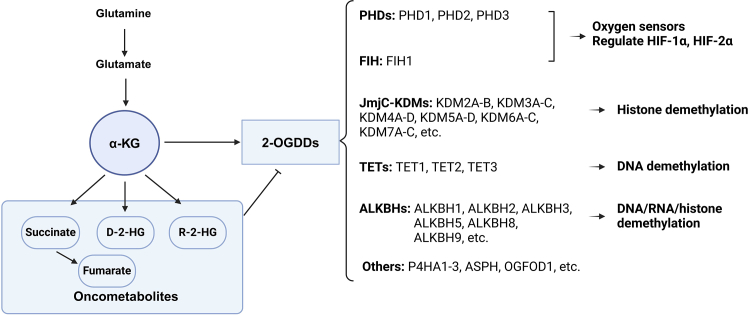
α-KG, oncometabolites, and 2-OGDD functions α-KG is an essential co-substrate that regulates 2-OGDD activity, requiring O_2_ and Fe^2+^ for enzymatic function. In contrast, succinate, fumarate, and 2-HG compete with α-KG, inhibiting 2-OGDDs. Succinate and fumarate, derived from α-KG, are TCA cycle intermediates. D-2-HG is produced from α-KG by mutant IDH, such as IDH1^R132H^, whereas R-2-HG arises from the promiscuous activities of MDH and LDH under acidic or hypoxic conditions. These metabolites structurally mimic α-KG and act as competitive inhibitors of 2-OGDDs. α-KG and oncometabolites mediate the crosstalk between metabolic reprogramming and epigenetic modifications. Abbreviation: 2-OGDDs or αKGDs, 2-oxoglutarate-dependent dioxygenases or α-KG-dependent dioxygenases; α-KG, α-ketoglutarate; ASPH, aspartate β-hydroxylase; ALKBH, AlkB homolog; D-2-HG, D-2-hydroxyglutarate; FIH, factor inhibiting hypoxia-inducible factor; HIF, hypoxia-inducible factor; LDH, lactate dehydrogenase; MDH, malate dehydrogenase; OGFOD1, 2-oxoglutarate and iron dependent dioxygenase 1; P4HA, collagen prolyl 4-hydroxylase α subunit; PHDs, prolyl-4-hydroxylase domain-containing proteins; R-2-HG, R-2-hydroxyglutarate. Created in BioRender. Sisi, Z. (2025) https://BioRender.com/b33y737.

Epigenetic modifications, including DNA methylation, histone methylation and acetylation, chromatin structure regulation, and RNA modification, serve as fundamental mechanisms regulating gene expression in various biological processes.^150^ Dysregulated epigenetic alterations driven by epigenetic enzymes are widely observed in cancers.^38^^,^^39^^,^^151^^,^^152^^,^^153^ Metabolites generated through tumor-associated metabolic reprogramming function as substrates, cofactors, or regulators of epigenetic enzymes, thereby influencing their activity and subsequent epigenetic modifications. For instance, α-KG-dependent JmjC-KDMs demethylate lysine residues in histones.^154^^,^^155^^,^^156^ Overexpression of GDH1 or α-KG supplementation reduces H3K9me3 and H3K27me3 levels, leading to increased expression of glial cell markers.^157^ Similarly, α-KG-dependent TET dioxygenase mediate DNA 5-methylcytosine (5mC) oxidation,^158^^,^^159^^,^^160^ while ALKBH5 and ALKBH9 regulate m^6^A demethylation in RNA.^161^^,^^162^^,^^163^ These epigenetic enzymes collectively modify histones, DNA, and RNA, thereby influencing gene expression and impacting tumorigenesis and cancer therapy (Figure 3).^39^^,^^149^^,^^161^^,^^162^

The interactions among α-KG, α-KG-derived oncometabolites, 2-OGDDs, and epigenetic modifications are intricate and exhibit cell-type specificity. For instance, in embryonic stem cells, α-KG modulates 5mC levels by regulating the expression of DNA methyltransferase (DNMT) 3A and 3B rather than TET activity.^164^ The GDH1/α-KG axis links metabolic reprogramming to epigenetic regulation, highlighting their coordinated influence on cell fate determination.

### GDH1 promotes redox homeostasis

Maintaining a delicate balance between the generation and elimination of reactive oxygen species (ROS) is essential for cellular homeostasis. This equilibrium is particularly critical in rapidly proliferating cells, where heightened metabolic activity leads to increased ROS production.^165^^,^^166^^,^^167^ GDH1 contributes to redox homeostasis through several distinct mechanisms. First, the conversion of glutamate to α-KG is coupled to NADPH production, which fuels antioxidant defense systems.^13^^,^^20^^,^^21^^,^^168^ Thioredoxin reductase (TRxR) utilizes NADPH to sustain thioredoxin (TRx) in its reduced state, facilitating H_2_O_2_ detoxification, while glutathione reductase (GR) employs NADPH to convert oxidized glutathione disulfide (GSSG) into reduced glutathione (GSH), thereby mitigating ROS levels.^21^ Second, GDH1-derived α-KG promotes fumarate accumulation, which directly binds to and activates GPx1, further enhancing antioxidant capacity.^3^ These studies highlight the crucial roles of GDH1, α-KG, and NADPH in maintaining the cellular redox homeostasis.

### GDH1-mediated ammonia production and recycle

Ammonia is generated as a byproduct of glutamate deamination by GDH1, as glutamate is converted to α-KG. While most ammonium is utilized as a nitrogen source for nucleotide biosynthesis or eliminated via the urea cycle, excessive ammonium promotes autophagy, which is associated with drug resistance and cancer progression.^30^^,^^169^^,^^170^ GDH1 also catalyzes the reductive amination of α-KG to generate glutamate under specific conditions.^44^^,^^45^^,^^46^^,^^47^^,^^48^^,^^49^ For instance, under glutamine-depleted conditions, ammonia can be utilized for glutamate production in a GDH2-dependent manner, thereby promoting cell proliferation in breast cancer (BC).^22^ As for poorly vascularized breast tumors, ammonia accumulation in the tumor microenvironment provides a direct substrate for glutamate synthesis, which subsequently fuels amino acid biosynthesis through GDH1/2 activity.^47^

## Progress on the roles of GDH1 in tumors

The functions of GDH1 in tumors are context-dependent, influenced by tumor pathology, microenvironment, and genetic alterations. Emerging evidence highlights its involvement in tumor progression through diverse regulatory mechanisms (Table 1).

**Table 1:** Progress on the roles of GDH1 in tumors

Disease	Types of GDH	Phenotypes	Molecule mechanisms	Reference
Glioblastoma	GDH1	Promote proliferation in cells with EGFR mutation	MEK/ERK/ELK1, EGFR, α-KG	Yang et al.^143^
	GDH1	Promote survival under glucose deprivation	α-KG, TCA	Yang et al.^51^
	GDH1	Promote proliferation and colony formation	α-KG, TCA, ROS	Zhang et al.^171^
	GDH1	Promote proliferation	α-KG, TCA	Miki et al.^172^
	GDH1	Promote survival under low glucose	α-KG, NF-κB, GLUT1	Wang et al.^19^
	GDH1	Promote proliferation	α-KG, KDM6A, H3K27me3, PDPK1, PI3K/AKT/mTOR	Yang et al.^173^
	GDH2	Promote growth in IDH1^R132H^ cells	2-HG, α-KG, TCA	Chen et al.^127^
	GDH2	Promote growth in IDH1^R132H^ cells	2-HG, α-KG, TCA	Waitkus et al.^128^
	GDH2	Inhibit proliferation, migration, invasion in cells without IDH1/IDH2 mutations	Cell cycle, mitochondrial functions and ROS	Franceschi et al.^174^
Pancreatic cancer	GDH1	Promote proliferation and migration	XLOC_006390/c-Myc	He et al.^175^
	GDH1	Not a principal modulator of of nitrogen scavenging and growth in KRAS^G12D^-mutant cells	–	Bott et al.^176^
	GDH1	Not a principal modulator of glutaminolysis and growth in KRAS-mutant cells	–	Son et al.^177^
	GDH1	Not a principal modulator of chemoresistance in KRAS-mutant cells	–	Mukhopadhyay et al.^178^
Lung cancer	GDH1	Promote proliferation and tumor growth	α-KG, fumarate, GPx1, redox homeostasis	Jin et al.^3^
	GDH1	Maintain survival under glucose deprivation	SIRT3, p62, autophagy	Hu et al.^50^; Hu et al.^179^
	GDH1	Promote proliferation and migration	STUB1, ubiquitin-mediated proteasomal degradation	Hu et al.^180^
	GDH1	Promote drug resistance (docetaxel) and metastasis	α-KG, ROS, Snail, EMT	Wang et al.^56^
	GDH1	Promote drug resistance (cisplatin)	Hypoxia, HIF-1α, α-KG	Jiang et al.^181^
	GDH1	Promote anoikis resistance and metastasis in LKB1-deficient cells	α-KG, CamKK2, AMPK	Jin et al.^54^
	GDH1	Promote anoikis resistance and metastasis	α-KG, CaMKK2, CaMKIV, CREB	Kang et al.^55^
Breast cancer	GDH1	Promote proliferation and tumor growth	α-KG, fumarate, GPx1, redox homeostasis	Jin et al.^3^
	GDH1	Promote anoikis resistance	EMT, α-KG, ROS	Farris et al.^182^
	GDH1/2	Promote growth and proliferation	Ammonia assimilation	Spinelli et al.^47^
Colorectal cancer	GDH1	Promote proliferation, migration, and invasion	STAT3, EMT	Liu et al.^183^
	GDH1	Promote survival under low glucose	Balance between ROS and energy production	Miyo et al.^184^
	GDH1	Promote proliferation and drug resistance (cisplatin and 5-Fu)	γH2AX, catalase, SOD1, GPx1, apoptosis	Chang et al.^53^
	GDH1	Promote proliferation	SIRT5, deglutarylation, α-KG, TCA	Wang et al.^185^
	GDH1	Promote survival under hypoxia	p300, HIF-1α, EGLN1, α-KG	Hu et al.^45^
Liver cancer	GDH1	Promote proliferation	ROS, apoptosis	Marsico et al.^186^
	GDH1	Promote survival under glucose deprivation	α-KG	Zhou et al.^31^
	GDH1	Promote survival under stress	α-KG, OXPHOS	Mi et al.^187^
	GDH1	Suppress proliferation, migration, invasion	ROS, p38/JNK MAPK, apoptosis	Zhao et al.^188^
	GDH1	Suppress growth and migration	AKT, IL-32	You et al.^189^
Kidney cancer	GDH1	Suppress proliferation and metastasis	PI3K/Akt/mTOR	Wang et al.^190^
	GDH1	Sensitize cells to amino acid deprivation	α-KG, KDMs, H3K9me3, H3K27me3, RP	Shao et al.^191^
	GDH1	Inhibit immunoevasion	MHC class I	Li et al.^192^

### Glioblastoma

Glioblastoma (GBM) is an aggressive glioma subtype in humans, with a median survival of 15 months.^193^ Genetic alterations in epidermal growth factor receptor (EGFR), present in ∼50% of GBM patients, contribute significantly to its aggressiveness.^194^ Yang et al. demonstrated that EGFR activated *GLUD1* transcription through the MEK/ERK/ELK1 pathway.^143^ MEK1 and ERK1/2 activation enhanced ELK1 phosphorylation at S383, facilitating its nuclear translocation and enrichment at the *GLUD1* promoter region to drive transcription. Metabolic adaptation to the low-glucose tumor environment also involves GDH1 regulation, with glucose deprivation markedly enhancing GDH1 activity in SF188 cells.^51^ Miki et al. also demonstrated that multiple GBM cell lines, including U87, U373, and two patient-derived cell lines, upregulated GDH1 expression under glucose-starved conditions.^172^ Pro-tumorigenic IL-11Rα overexpression correlated with elevated GDH1 expression in GBM patients, but the interactions require further investigation.^195^

GDH1 is generally considered protumoral in GBM, especially under glucose-limiting conditions. Glutaminolysis supports impaired glucose metabolism by providing energy and metabolites, a process that is dispensable in glucose-utilizing cells but becomes critical under conditions with inhibited glycolysis, such as glucose deprivation, 2-deoxyglucose (2-DG) treatment, or Akt inhibition.^51^ GDH1 in GBM has been reported to maintain the ongoing TCA cycle and provide precursors for lipid, protein, and nucleic acid synthesis.^41^^,^^51^^,^^52^ GDH1 overexpression promoted proliferation and colony formation in U251 cells,^171^ while *GLUD1* knockdown or pharmacological inhibition of GDH1 using epigallocatechin gallate (EGCG) sensitized GBM cells to glucose deprivation.^51^ Supplementation with the cell-permeable dimethyl-α-KG (DM-α-KG) restored GBM cell proliferation suppressed by *GLUD1* silencing.^143^ Inhibiting the glutaminolysis pathway, including GDH1, may be an effective strategy for GBM characterized by high glutaminolysis-related protein expression.^172^

The protumoral role of GDH1 extends beyond ATP production and precursor synthesis. Wang et al. revealed that α-KG produced by GDH1 directly regulated the nuclear factor κB (NF-κB) pathway, promoting glucose uptake and GBM development.^19^ Under low-glucose conditions, GDH1 generated a local source of α-KG, which bound to IκB kinase β (IKKβ) and activated NF-κB signaling, resulting in *GLUT1* upregulation to enhance glucose transport. This process was crucial for the survival of U87 and U251 cells under low-glucose conditions. α-KG produced by GDH1 modulated KDM6A, a 2-OGDD involved in histone demethylation, reducing the suppressive histone modification H3K27me3.^173^ Consequently, the transcription of phosphoinositide-dependent kinase-1 (PDPK1) was increased, leading to PI3K/AKT/mTOR pathway activation. This modulation was essential for GBM cell proliferation and brain tumorigenesis, even under high-glucose conditions.

The presence of GDH2 in GBM combined with the glutamate-rich environment in the brain, adds complexity to the scenario. The IDH1^R132H^ mutation redirects metabolic flux, generating high concentrations of D-2-HG from α-KG.^196^ IDH1^R132H^ is frequently present in most secondary GBM and has been recognized as the most common initiating event.^127^ Interestingly, IDH1^R132H^ inhibited growth, accompanied by reduced metabolic flux from glucose and glutamine to lipids in glioma progenitor cells. Overexpression of GDH2, but not GDH1, reversed the impact of the IDH1^R132H^ mutation on metabolic flux and promoted tumor growth.^127^^,^^128^ Compared with GDH1, GDH2 bears a lower optimal pH, enabling it to efficiently process the excessive glutamate flux in the brain and align with the acidified intracellular conditions induced by D-2-HG, whereas the activity of GDH1 is greatly inhibited. Extracellular glutamate might also contribute to redox homeostasis through GDH2, potentially increasing GSH/GSSG and NADPH/NADP^+^ ratios to mitigate IDH1^R132H^-mediated suppression.^197^

GDH2 exhibited a distinct role in GBM with wild-type IDH1/2, where elevated *GLUD2* expression correlated with better clinical outcome.^174^ It was reported that GDH2 overexpression inhibited GBM cell proliferation, migration, invasion, and colony formation. These roles were attributed to GDH2 regulation of cyclin D1 and cyclin E expression, influencing cell cycle progression, mitochondrial function, and ROS production. Notably, the metabolic heterogeneity of glutamine in GBM underscores the critical need for precisely modulating GDH1 and GDH2 in therapeutic strategies.

### Pancreatic cancer

Pancreatic ductal adenocarcinoma (PDAC) accounts for over 90% of pancreatic cancers.^198^^,^^199^ It is highly aggressive, with a poor prognosis and significant resistance to radiotherapy, chemotherapy, and immunotherapy, leading to a 5-year survival rate of less than 10%. PDAC cells are highly dependent on glutamine for proliferation and redox balance.^176^^,^^177^^,^^200^^,^^201^ Additionally, glutamine deprivation increases the sensitivity of PDAC cells to chemotherapeutics, such as gemcitabine, 5-fluorouracil, oxaliplatin, and cisplatin.^176^^,^^178^^,^^202^ Despite the dependency of PDAC cells on glutamine, the expression and role of GDH1 in PDAC may vary under different conditions.

Roux et al. reported increased mRNA expression of both *GLUD1* and *GLUD2*, along with elevated GDH1/2 activity, in several PDAC cell lines (excluding PANC-1), compared to non-PDAC controls, supporting nucleotide and ATP production.^203^ They also observed a reduction in blood glutamine as the tumor progressed from pancreatic intraepithelial lesions to invasive PDAC. Long non-coding RNAs (lncRNAs) are involved in key biological processes by interacting with macromolecules (e.g., miRNA, mRNA, and proteins), such as forming lncRNA-protein complexes to regulate protein activity or stability.^204^^,^^205^ He et al. demonstrated that lncRNA XLOC_006390 bound to c-Myc, protecting it from ubiquitination and degradation, thereby activating *GLUD1* transcription in PDAC cells.^175^ Higher *GLUD1* expression was detected in pancreatic tumors compared to paired adjacent tissues, as well as in recurrent pancreatic cancers compared to non-recurrent cases. These studies suggest a potential positive correlation between GDH1 and PDAC progression.

It is important to note that genetic mutations, such as the commonly harbored KRAS mutations, drive oncogenesis and progression of pancreatic cancers.^206^^,^^207^ KRAS-activating mutations have been reported to enhance glutamine metabolism by increasing uptake and reprogramming glutaminolysis.^177^^,^^178^^,^^208^ Several studies suggested that the role of GDH1 in PDAC with KRAS mutations might not be as significant. A comparative proteomic analysis revealed that in PANC-1 cells harboring the KRAS^G12D^ mutation, GDH1 expression did not show an evident increase compared to normal pancreatic duct cells.^209^ Interestingly, three glutamine-utilizing enzymes—cytidine triphosphate synthase (CTPS), guanine monophosphate synthetase (GMPS), and asparagine synthetase (ASNS)—were found to be significantly upregulated in PANC-1 cells, indicating that glutamine was primarily consumed as a nitrogen donor in nucleotide and amino acid biosynthesis, rather than as a mitochondrial substrate. This hypothesis was supported by a study showing that co-treatment with α-KG and non-essential amino acids (NEAA) rescued glutamine-deprived PDAC cells harboring the KRAS^G12D^ mutation in a manner dependent on glutamate ammonia ligase (GLUL), which was essential for α-KG-coupled glutamine biosynthesis and subsequent nitrogen anabolic processes.^176^ Additionally, inhibition of GOT and GPT, but not GDH1, led to reduced cell growth, suggesting that under glutamine-deprived conditions, PDAC cells might scavenge nitrogen from sources like alanine and aspartate to transaminate α-KG into glutamate for glutamine synthesis, bypassing GDH1. Son et al. acknowledged that the KRAS mutation in PDAC cells reprogrammed glutamine metabolism toward a distinct GDH1-independent pathway.^177^ They found that oncogenic KRAS mutation was positively correlated with GOT1 expression and negatively correlated with GDH1 expression. The combination of α-KG and NEAA, but not α-KG alone, rescued PDAC cell proliferation upon glutamine deprivation. GDH1 knockdown or inhibition had no effect on cell growth, whereas pan-transaminase inhibition or GOT1 knockdown significantly inhibited the growth of multiple PDAC cell lines under glutamine deprivation. GOT1 converts glutamine-derived aspartate into oxaloacetate (OAA), which is then converted into malate by malate dehydrogenase 1 (MDH1) and subsequently oxidized by malic enzyme 1 (ME1) into pyruvate, generating reducing power in the form of NADPH. Thus, GOT1 knockdown led to an accumulation of glutamine-derived aspartate, as well as a decrease in OAA and the GSH/GSSG ratio. Suppression of MDH1 and ME1 significantly impaired PDAC cell survival, which was rescued by GSH replenishment or N-acetylcysteine. These results underscored the critical role of GDH1-independent glutamine metabolism in maintaining redox homeostasis in PDAC with KRAS mutations. Other studies also supported the role of GOT1 in promoting PDAC progression.^210^^,^^211^ NRF2, reported as another downstream factor of KRAS mutations in PDAC cells, drove preferential activation of GOT1 over GDH1, contributing to chemoresistance to multiple drugs.^178^ These studies demonstrate the dependency of PDAC cells on glutamine; however, due to the high mutational heterogeneity in PDAC, glutamine metabolism may predominantly be mediated by enzymes other than GDH1.

### Lung cancer

Lung cancer (LC) is the leading cause of cancer-related deaths globally.^212^^,^^213^ A major challenge in treating LC is its high propensity for metastasis, with a 5-year survival rate for metastatic cases of only ∼3%. Similar to other tumors, increasing evidence over the past years has shown that LC cells are addicted to glutamine to fulfill their metabolic needs.^13^^,^^214^^,^^215^ The dependency of LC cells on glutamine for growth and survival has been well documented in various studies.^216^^,^^217^^,^^218^^,^^219^ As a critical enzyme in glutaminolysis, the role of GDH1 in promoting tumor progression has also been validated in LC.^3^^,^^50^^,^^180^ Jin et al. reported that GDH1 expression was significantly increased in tumors from patients with advanced-stage LC compared to normal lung tissues.^3^ Sirtuin 4 (SIRT4), a negative regulator of GDH1, was reduced in LC, which might contribute to GDH1 upregulation.^220^^,^^221^ GDH1, rather than GOT2 or GPT2, was the predominant enzyme responsible for converting glutamate to α-KG in H1299 cells.^3^ GDH1 knockdown significantly decreased cell proliferation, particularly under oxidative stress and low glucose conditions. Further studies revealed that GDH1 regulated intracellular α-KG levels, leading to increased fumarate, which activated GPx1 in LC cells and contributed to redox balance. Hu et al. reported that GDH1-K84 deacetylation by SIRT3 under glucose deprivation promoted the formation of highly active GDH1 hexamers.^50^ This modification also induced GDH1 cytoplasmic localization, where it interacted with p62 and inhibited autophagic cell death, thereby maintaining survival under glucose deprivation. Enhanced protein stability via the proteasome degradation pathway is another key mechanism regulating GDH1 in LC.^180^ K503 is the main ubiquitination site of GDH1 by the E3 ligase STIP1 homology and U-box-containing protein 1 (STUB1). Overexpression of STUB1 reduced the enhanced proliferation mediated by GDH1 overexpression, while inhibiting GDH1 ubiquitination at K503 promoted tumorigenicity in LC cells.

Beyond its role in tumor development and progression, GDH1 is also involved in drug resistance and metastasis. Six months of cisplatin- or carboplatin-based chemotherapy elevated glutamate levels in both the serum and peripheral blood mononuclear cells (PBMCs) of LC patients.^222^ Additionally, GDH1/2 activity significantly increased in PBMCs but not in the serum. Gefitinib treatment significantly reduced intracellular glutamine content in A549 cells, but not in PC-9 cells, which are considered gefitinib-resistant and gefitinib-sensitive, respectively.^218^ Docetaxel (DTX) treatment dose-dependently upregulated GDH1.^56^ Moreover, an increased glutamate-to-glutamine ratio and elevated GDH1 expression were observed in DTX-resistant A549 cells. These studies highlighted the associations of chemotherapy with glutaminolysis and GDH1 regulation, which might contribute to acquired drug resistance.^56^ Hypoxia is a hallmark of solid tumors, and hypoxia adaptation in cancer cells is regarded as a driving force in drug resistance.^223^^,^^224^ Hypoxia upregulated HIF-1α, which bound to the *GLUD1* promoter and activated its transcription in LC cells.^181^ Hypoxia-induced increases in glutamate flux to α-KG and ATP/ADP ratio were blocked by GDH1 knockdown, highlighting its critical role in metabolic reprogramming upon hypoxia stress. Notably, GDH1 knockdown enhanced cisplatin-induced toxicity under hypoxia, while overexpression attenuated this response. These results suggested that the HIF-1α-GDH1 pathway contributed to cisplatin resistance in LC.

As a metastasis barrier, cells typically undergo anoikis, a form of cell death caused by the loss of contact with the extracellular matrix or neighboring cells.^225^ Anoikis resistance enables cancer cells to survive detachment from primary sites and spread through the circulatory and lymphatic systems, making it a critical prerequisite for tumor metastasis.^226^^,^^227^ GDH1 is the predominant isoform in various LC cell lines and is commonly upregulated under metastatic conditions associated with cell detachment.^54^ The GDH1 product α-KG bound to calcium/calmodulin-dependent protein kinase kinase 2 (CamKK2), which facilitated the recruitment and activation of AMP-activated protein kinase (AMPK). This activation contributed to energy production, which conferred anoikis resistance in liver kinase B1 (LKB1)-deficient cells, where CamKK2 played a dominant role in AMPK activation over LKB1. Kang et al. reported another mechanism by which GDH1 mediates anoikis resistance in LC.^55^ GDH1 inhibition by R162 induced mild anoikis, while dual inhibition of GDH1 and ribosomal S6 kinase 2 (RSK2) synergistically suppressed invasion and enhanced anoikis sensitivity, particularly in tumors harboring EGFR-activating or EGFR inhibitor-resistant mutations. Mechanistically, EGFR phosphorylated GDH1 at Y135, activating it and increasing α-KG production. α-KG activated CamKK2, which sequentially increased the activity of Calcium/calmodulin-dependent protein kinase IV (CaMKIV). The synergistic interaction between CaMKIV and RSK2 enhanced cAMP response element-binding protein (CREB) activity, ultimately promoting invasion and anoikis resistance in LC cells. α-KG replenishment restored the reduced CREB activity and anoikis resistance caused by GDH1 knockdown, highlighting a crucial role of GDH1 in tumor metastasis.^55^

The epithelial-mesenchymal transition (EMT) is also a pivotal factor mediating tumor invasion and drug resistance.^228^^,^^229^ It is a process in which cells transition from epithelial characteristics, such as stable adhesion and apical-basal polarity, to mesenchymal traits, including enhanced motility, invasiveness, and resistance to apoptosis. Wang et al. demonstrated the involvement of GDH1-dependent glutamine metabolism reprogramming in promoting EMT-mediated metastasis in DTX-resistant LC cells.^56^ Metabolomics assays revealed impaired glycolysis and an increased glutamate-to-glutamine ratio in A549/DTX cells compared to A549 cells. Additionally, there was a concomitant strengthening of the TCA cycle, along with an increased NADH/NAD^+^ ratio and ATP level. LC-MS-based isotope tracer analysis using [U-^13^C_5_]-glutamine demonstrated enhanced glutamine catabolism, with significantly increased m+5 labeling of glutamate and α-KG, as well as other m+4 labeled TCA cycle intermediates. Moreover, GDH1 was significantly upregulated in A549/DTX cells. These results indicated a greater dependence on glutamine catabolism for energy supply in DTX-resistant cells. GDH1 inhibition or silencing suppressed ROS accumulation and the expression of the EMT transcription factor Snail, as well as EMT-driven cell migration and invasion.^56^ This effect was completely abolished by α-KG repletion. Moreover, GDH1 inhibition enhanced sensitivity to DTX in LC cells. ROS are regarded as a 'double-edged sword' in oncogenesis, with their effects contingent on the activated signaling pathways.^230^^,^^231^ ROS are reported to act upstream in regulating Snail and EMT through various mechanisms, including the PKC-MAPK and NF-κB signaling pathways.^232^^,^^233^ GDH1-mediated ROS accumulation is considered a key driver of Snail overexpression and EMT in DTX-resistant LC cells.^56^

Collectively, these studies highlight the pivotal role of GDH1 and its product α-KG in driving proliferation, migration, and drug resistance in LC.

### Breast cancer

BC manifests inherent heterogeneity, encompassing diverse histological and molecular attributes.^234^^,^^235^ Understanding this molecular heterogeneity is crucial for developing appropriate treatments. The roles of GDH1 vary across BC subtypes. The catalytic activity of GDH1/2 was significantly elevated in both the serum and tumor tissues of BC patients, compared to control serum and non-cancerous tissues, respectively.^236^ However, the study lacked detailed information on the molecular subtypes of BC. The mRNA and protein expression of GDH1 were elevated in luminal A/B (ER^+^) BC, compared to triple-negative breast cancer (TNBC), but the level of GDH1 in HER2^+^ tumors remained controversial across different studies.^237^^,^^238^

In the TNBC cell line MDA-MB-231, GDH1 was the primary enzyme responsible for converting glutamate to α-KG, and GDH1 knockdown led to a significant decrease in cell proliferation.^3^ Further experiments revealed that GDH1 regulated glutaminolysis and intracellular α-KG levels, leading to increased fumarate, which directly bound to and activated GPx1 to scavenge ROS. Farris et al. found that GDH1 expression was increased during EMT in human mammary epithelial cells.^182^ They also observed that high GDH1 expression and DM-α-KG protected cells against anoikis by neutralizing ROS. Beyond maintaining redox homeostasis, Spinelli et al. demonstrated that GDH1 facilitated reductive amination in ER^+^ BC.^47^ The ^15^N(amide)-glutamine liberated ^15^NH_3_ through GS activity and was used to investigate the fate of glutamine-derived ammonia. The ER^+^ MCF7 cells, which express both GDH1 and GDH2 isoforms, were treated with ^15^N(amide)-glutamine. The ^15^N-isotopologues were detected in proline, aspartate, branched-chain amino acids (BCAA), and glutamate, which have no previous biosynthetic connection to the amide-nitrogen on glutamine. The abundance of ^15^N-isotopologues, including glutamate and its downstream metabolites (proline, aspartate), significantly decreased in cells with GDH1/2 depletion. Notably, the urea cycle intermediates (ornithine, citrulline, argininosuccinate, and arginine) remained unlabeled, regardless of GDH1/2 expression. These findings demonstrated that GDH1/2, rather than the urea cycle, catalyzed ammonia assimilation to produce glutamate and downstream amino acids, thereby maximizing nitrogen utilization in ER^+^ BC.^47^

Contradictory roles of GDH1 in BC have been documented. Craze et al. reported that elevated GDH1 was correlated with good overall patient outcomes but not in any specific molecular subtypes, and GDH1 expression was associated with better outcomes in triple-negative patients treated with chemotherapy.^237^ This study highlights the influence of molecular subtypes on BC metabolism. However, additional cohorts are needed for further investigation to validate the role of GDH1 in heterogeneous BC subtypes.

The role of GDH1 varies across BC molecular subtypes and warrants further investigation. GDH1 is significantly upregulated in ER^+^ BC, where it may promote tumorigenesis through both forward and reverse reactions, such as facilitating glutamate deamination to maintain redox balance or catalyzing ammonia assimilation to optimize nitrogen utilization.

### Colorectal cancer

Colorectal cancer (CRC), the third most diagnosed cancer and the second leading cause of cancer-related deaths globally, continues to pose a significant global health challenge.^239^^,^^240^ GDH1 has emerged as a pivotal player in promoting CRC progression.

Notably, GDH1 expression is upregulated in both CRC cell lines and tissues, correlating with CRC metastasis and poor prognosis.^183^^,^^184^ GDH1 was reported to promote cell proliferation, migration, and invasion in CRC cells.^183^ GDH1 overexpression induced signal transducer and activator of transcription 3 (STAT3) phosphorylation at Y705, upregulated Vimentin and ZEB1 expression, while decreasing E-cadherin expression. Inhibition of STAT3 with AG490 significantly blocked the effect of GDH1 overexpression, suggesting that GDH1 promoted CRC progression via STAT3-mediated EMT induction. Miyo et al. demonstrated that GDH1 was significantly increased in CRC cells resistant to glucose deprivation, helping retain TCA cycle activity and adapt to nutritional stress.^184^ In contrast, GDH1 knockdown markedly decreased cell growth. GDH1 expression was high in both wild-type and multidrug-resistant CRC cells.^53^ Inhibition of GDH1 resulted in decreased expression of gamma-H2A histone family member X (γH2AX), catalase, superoxide dismutase 1 (SOD1), and GPx1, increasing ROS accumulation and cell death.

Post-translational modifications represent a mechanism to modulate GDH1 activity and control tumor metabolism. Mitochondrial Sirtuin 5 (SIRT5) contributed to a malignant phenotype in CRC.^185^ Specifically, SIRT5 reduced lysine glutarylation of GDH1 at K545, thereby activating GDH1, which in turn enhanced the glutamate-to-α-KG conversion and TCA cycle flux. GDH1 acetylation has been implicated in enhancing HIF-1α stability to promote CRC progression under hypoxia.^45^ Hu et al. found that CRC cells lacking GDH1 were more sensitive to hypoxia, but DM-α-KG supplementation did not rescue the effects of GDH1 depletion on HIF-1α levels and cell viability, suggesting a mechanism beyond glutaminolysis under hypoxic conditions. GDH1 was acetylated at K503 and K527 by p300 in response to hypoxia. Acetylation at K503 increased the affinity of GDH1 for α-KG, promoting reversed glutaminolysis and generating glutamate. In contrast, acetylation at K527 facilitated the formation of a GDH1 complex with EGLN1/HIF-1α. This reversed GDH1 activity likely consumed α-KG to produce glutamate, reducing α-KG concentrations around the GDH1/EGLN1/HIF-1α complex. EGLN1 (also known as PDH2, a 2-OGDD) activity was significantly influenced by its cofactor α-KG and closely linked to HIF-1α stability. Thus, acetylation of GDH1 at K503 and K527 under hypoxia stabilized HIF-1α, leading to its accumulation. The function of HIF-1α as a key regulator in promoting CRC progression has been confirmed by numerous studies.^241^^,^^242^^,^^243^^,^^244^

### Liver cancer

Hepatocellular carcinoma (HCC) is among the most lethal cancers, with its pathogenesis intricately linked to metabolic dysregulation.^245^^,^^246^ HCC relies on glutamine for survival and proliferation, emphasizing the pivotal role of enzymes in the glutamine metabolism pathway, such as GDH1, in its progression.^12^^,^^31^ GDH1 is the specific isoform expressed in the liver, with its expression being the highest among all tissues.^18^^,^^27^ As hepatocytes are exposed to various concentrations of glutamate and ammonia, GDH1 is believed to function in both oxidative deamination and reductive amination directions.^27^^,^^85^^,^^86^ Thus, the role of GDH1 in HCC may vary under different conditions.

Serum GDH1/2 levels at the time of liver transplantation independently predicted the probability of tumor recurrence and microvascular invasion post-surgery, indicating a possible positive role of GDH1/2 in promoting HCC progression.^247^^,^^248^ Marsico et al. reported that *GLUD1* expression was higher in HepG2 cells compared to other tumor cell lines, and also higher in liver cancers relative to normal liver tissues and hepatocytes.^186^ *GLUD1* significantly reduced cell proliferation in HepG2 cells but increased proliferation in human hepatocytes, indicating distinct roles of GDH1 in HCC and normal hepatocytes. A study by Zhou et al. revealed that GDH1 played a crucial role in HCC cell survival and proliferation under glucose starvation, while the glutaminolysis pathway in HCC cells was altered in response to varying glucose conditions.^31^ GDH1 knockdown or inhibition significantly reduced HCC cell proliferation under glucose limitation, but had no effect under high-glucose conditions. In contrast, GOT silencing or inhibition had no impact under low-glucose conditions, while it suppressed proliferation in high-glucose conditions. Upon glucose starvation, TCA cycle intermediates such as succinate, citrate, *cis*-aconitate, and oxaloacetate decreased more significantly after GDH1 knockdown. These results suggested that GDH1-mediated glutamine anaplerosis supported TCA cycle flux in response to glucose limitation, positioning it as a potential therapeutic target in low-glucose HCC tissues.^31^ Inhibition of bromodomain and extra-terminal domain (BET) decreased glycolytic gene expression but upregulated GDH1, which generated α-KG for the TCA cycle and OXPHOS.^187^ Targeting GDH1 was synthetically lethal in combination with BET inhibition in HCC cells.

Contradictory roles of GDH1 have been reported recently. GDH1 expression was significantly decreased in HCC tumors and was associated with poor prognosis.^188^^,^^189^ Zhao et al. reported that GDH1 overexpression markedly suppressed HCC cell proliferation, migration, invasion, and tumor growth both *in vitro* and *in vivo*, whereas GDH1 knockdown accelerated HCC progression.^188^ They demonstrated that GDH1 overexpression enhanced mitochondrial respiration and ROS production, leading to p38/JNK MAPK pathway activation and mitochondrial apoptosis. N-acetylcysteine (NAC) treatment reduced cellular ROS, suppressed p38/JNK MAPK pathway activation, and mitigated apoptosis induced by GDH1 overexpression. You et al. also demonstrated that GDH1 silencing enhanced HCC cell growth and migration.^189^ GDH1 silencing activated AKT and upregulated cytokines that facilitated HCC cell growth and migration. The hepatitis B virus (HBV) X protein (HBX reduced GDH1 protein stability by promoting its ubiquitination, mediated by LIM and SH3 protein 1 (LASP1) and E3 ubiquitin ligase synoviolin (SYVN1), indicating the possible role of GDH1 in contributing to HBV-induced HCC.^189^

HCC may exhibit distinct glutamine metabolic profiles under different conditions, and the precise role of GDH1 in HCC requires further *in vivo* investigation. Notably, GDH1 has been identified as a negative regulator of HCC cell survival under glucose-limited conditions.

### Kidney cancer

Kidney renal clear cell carcinoma (KIRC) is the most common and aggressive subtype of renal cell carcinoma (RCC).^249^^,^^250^ GDH1 is the primary metabolic enzyme for α-KG production in KIRC cells, with GDH1 depletion reducing intracellular α-KG levels by 60%–70%.^191^

Remarkably, unlike many other cancers, GDH1 primarily functions as a negative regulator in KIRC. Increased methylation of the *GLUD1* promoter in KIRC tissues was linked to reduced GDH1 expression, which correlated with poorer survival and resistance to tyrosine kinase inhibitors (TKIs).^190^ Downregulation of GDH1 in KIRC cells activated the PI3K/Akt/mTOR signaling pathway, promoting cell proliferation and migration, although the underlying mechanism was not specified. It also contributed to the formation of an immunosuppressive tumor microenvironment by enhancing the infiltration of dysfunctional immune cells. Another study by Shao also supported the tumor-suppressive role of GDH1 in KIRC.^191^ Increased GDH1 expression sensitized KIRC cells to amino acid deprivation, whereas reduced GDH1 expression had the opposite effect. GDH1 downregulation caused a significant reduction in intracellular α-KG levels, resulting in decreased KDM activity, which in turn increased H3K9 and H3K27 methylation at ribosomal protein (RP) gene promoters and suppressed RP gene expression. This process ultimately promoted nutrient preservation to support KIRC cell survival. Clinical samples from KIRC patients at stages II-IV revealed an inverse correlation between GDH1 and ring finger protein 213 (RNF213), an E3 ligase of GDH1, with elevated RNF213 expression and decreased GDH1 associated with poor prognosis.^191^ Under nutrient-rich conditions, GDH1 maintained the expression of RP genes through its enzymatic activity. Early-stage KIRC (stage I) typically had an abundant nutrient supply, and KIRC patients with lower GDH1 expression exhibited better survival, suggesting an aggressive role of GDH1 in nutrient-rich conditions.

Low GDH1-mediated α-KG production was associated with immunoevasion in RCC.^192^ In contrast, elevated α-KG levels modulated broad-spectrum histone demethylation and promoted the transcriptional upregulation of genes encoding basic components of MHC class I molecules. The combination of α-KG supplementation and PD-1 blockade improved therapeutic efficacy and prolonged survival compared to monotherapy.

These studies demonstrate that, except in early-stage KIRC, GDH1 acts as a suppressor, and GDH1 inhibitors should be used with caution in KIRC.

## Identification of GDH1 inhibitors

Several strategies can be employed to inhibit GDH1, such as reducing its protein level or inhibiting its enzymatic activity. Inside cells, GDH1 is allosterically regulated by various metabolites, which bind non-covalently to the enzyme. The allosteric activators include ADP and leucine, while the inhibitors comprise GTP, ATP, NADH, and palmityl-CoA.^23^^,^^104^^,^^114^^,^^251^ In addition to metabolites, the short-chain 3-hydroxyacyl-CoA dehydrogenase (SCHAD) has been shown to interact with GDH1 and inhibit its activity.^252^^,^^253^ GDH1 activity is also regulated by covalent modifications, including ADP-ribosylation by SIRT4, a mitochondrial ADP-ribosyltransferase, which uses NAD^+^ to modify the enzyme within the mitochondria.^254^ Apart from these endogenous metabolites, a number of studies have identified other compounds capable of inhibiting GDH1, as listed in Table 2. Among them, several inhibitors have been studied for their therapeutic effects in cancer (Table 3).

**Table 2:** Identification of GDH inhibitors

Inhibitors	GDH	Activity^a^	Reference
Purpurin	Human GDH1	Ki 1.9 μM	Jin et al.^3^
R162	Human GDH1	Ki 28.6 μM	Jin et al.^3^
Epigallocatechin gallate (EGCG)	Human GDH1	IC_50_ 1.94 μM	Chang et al.^255^
	Bovine GDH	IC_50_ ∼0.3 μM	Li et al.^256^
	Bovine GDH	IC_50_ 0.5 μM	Li et al.^257^
	Bovine GDH	IC_50_ 0.7 μM	Zhu et al.^258^
	*Escherichia coli* GDH	IC_50_ 0.045 nM	Zhang et al.^259^
	*Escherichia coli* GDH	IC_50_ 0.05 μM	Zhu et al.^258^
	*Escherichia coli* GDH	IC_50_ 0.56 μM	Hou et al.^260^
Epicatechin gallate (ECG)	Bovine GDH	IC_50_ ∼300 nM	Li et al.^256^
	Bovine GDH	IC_50_ 0.5 μM	Li et al.^257^
Decursin (DN)	Human GDH1	IC_50_ 1.035 μM	Chang et al.^255^
Decursinol angelate (DA)	Human GDH1	IC_50_ 1.432 μM	Chang et al.^255^
Ebselen	Human GDH^b^	IC_50_ 2.4 μM	Hou^260^
	Human GDH^b^	IC_50_ 2.4 μM	Ruan et al.^261^
	Bovine GDH	IC_50_ ∼1 μM	Zhu et al.^258^
	*Escherichia coli* GDH	IC_50_ ∼0.2 μM	Zhu et al.^258^
	*Escherichia coli* GDH	IC_50_ 0.96 μM	Hou et al.^260^
	*Escherichia coli* GDH	IC_50_ 0.213 μM	Zhang et al.^259^
	*Escherichia coli* GDH	IC_50_ 0.36 μM	Yu et al.^262^
Propylselen	Human GDH^b^	IC_50_ 1.4 μM	Hou et al.^260^
	*Escherichia coli* GDH	IC_50_ 0.68 μM	Hou et al.^260^
Hexylselen (CPD 3B)	Human GDH^b^	IC_50_ 0.8 μM	Ruan et al.^261^
	Bovine GDH	IC_50_ 0.8 μM	Zhu et al.^258^
	*Escherichia coli* GDH	IC_50_ 0.3 μM	Zhu et al.^258^
CPD 1A	Bovine GDH	IC_50_ 0.8 μM	Zhu et al.^258^
	*Escherichia coli* GDH	IC_50_ 0.2 μM	Zhu et al.^258^
CPD 1B	Bovine GDH	IC_50_ 1.5 μM	Zhu et al.^258^
	*Escherichia coli* GDH	IC_50_ 0.2 μM	Zhu et al.^258^
CPD 2B	Bovine GDH	IC_50_ 1.3 μM	Zhu et al.^258^
	*Escherichia coli* GDH	IC_50_ 0.2 μM	Zhu et al.^258^
CPD 4B	Bovine GDH	IC_50_ 20 μM	Zhu et al.^258^
	*Escherichia coli* GDH	IC_50_ 0.5 μM	Zhu et al.^258^
CPD 5B	Bovine GDH	IC_50_ 1.6 μM	Zhu et al.^258^
	*Escherichia coli* GDH	IC_50_ 0.4 μM	Zhu et al.^258^
CPD 6B	Bovine GDH	IC_50_ 13 μM	Zhu et al.^258^
	*Escherichia coli* GDH	IC_50_ 2.3 μM	Zhu et al.^258^
CPD2	*Escherichia coli* GDH	IC_50_ 3.65 μM	Yu et al.^262^
CPD3	*Escherichia coli* GDH	IC_50_ 2.26 μM	Yu et al.^262^
CPD3	*Escherichia coli* GDH	IC_50_ 1.22 μM	Yu et al.^262^
Canagliflozin	Bovine GDH	IC_50_ > 50 μM	Secker et al.^263^
Diethylstilbestrol (DES)	Human GDH1	IC_50_ 1.67 μM	Borompokas et al.^264^
	Human GDH2	IC_50_ 0.08 μM	Borompokas et al.^264^
	Bovine GDH	IC_50_ > 2 μM	Yielding^265^
	Bovine GDH	IC_50_ 1.7 μM	Li^257^
Estradiol	Human GDH1	IC_50_ 26.94 μM	Borompokas et al.^264^
	Human GDH2	IC_50_ 1.53 μM	Borompokas et al.^264^
	Bovine GDH	IC_50_ > 35 μM	Yielding and Tomkins^265^
Estriol	Human GDH1	IC_50_ 144.77 μM	Borompokas et al.^264^
	Human GDH2	IC_50_ 11.34 μM	Borompokas et al.^264^
Progesterone	Human GDH1	IC_50_ 118.78 μM	Borompokas et al.^264^
	Human GDH2	IC_50_ 12.31 μM	Borompokas et al.^264^
	Bovine GDH	IC_50_ 72 μM	Yielding and Tomkins^265^
Pregnenolone	Human GDH1	IC_50_ 104.3 μM	Spanaki et al.^61^
	Human GDH2	IC_50_ 11.7 μM	Spanaki et al.^61^
Dehydroepiandrosterone (DHEA)	Human GDH1	IC_50_ 216.0 μM	Spanaki et al.^61^
	Human GDH2	IC_50_ 7.2 μM	Spanaki et al.^61^
Dihydrotestosterone (DHT)	Human GDH1	IC_50_ 493.5 μM	Spanaki et al.^61^
	Human GDH2	IC_50_ 49.4 μM	Spanaki et al.^61^
Corticosterone	Human GDH1	IC_50_ 1.1 μM	Spanaki et al.^61^
	Human GDH2	IC_50_ 2.1 μM	Spanaki et al.^61^
Quercetin	Bovine GDH	IC_50_ 8 μM	Marsico et al.^186^
Permethylated anigopreissin A (PAA)	Bovine GDH	IC_50_ 12 μM	Marsico et al.^186^
3-*O*-caffeoylquinic acid (3-CQA)	Bovine GDH	IC_50_ 52.0 μM	Domith et al.^266^
4-*O*-caffeoylquinic acid (4-CQA)	Bovine GDH	IC_50_ 44.4 μM	Domith et al.^266^
5-*O*-caffeoylquinic acid (5-CQA)	Bovine GDH	IC_50_ 84.5 μM	Domith et al.^266^
Caffeic acid	Bovine GDH	IC_50_ 158.2 μM	Domith et al.^266^
Chloroquine	Bovine GDH	IC_50_ 697.0 μM	Domith et al.^266^
	Human GDH1	IC_50_ 50 μM	Choi et al.^267^
	Human GDH2	IC_50_ 140 μM	Choi et al.^267^
Hexachlorophene (HCP)	Bovine GDH	IC_50_ 1.7 μM	Li et al.^257^
	Bovine GDH	IC_50_ 3.9 μM	Li et al.^268^
	*Tetrahymena* GDH	IC_50_ 1.9 μM	Li et al.^268^
	*Escherichia coli* GDH	IC_50_ 12 μM	Li et al.^268^
Bithionol (BTH)	Bovine GDH	IC_50_ 5.5 μM	Li et al.^257^
	Bovine GDH	IC_50_ 4.8 μM	Li et al.^268^
	*Tetrahymena* GDH	IC_50_ 5.9 μM	Li et al.^268^
	*Escherichia coli* GDH	IC_50_ 17 μM	Li et al.^268^
GW5074	Bovine GDH	IC_50_ 1.5 μM or 2.6 μM	Li et al.^257^
	Bovine GDH	IC_50_ 1.5 μM	Li et al.^268^
	*Tetrahymena* GDH	IC_50_ 2.6 μM	Li et al.^268^
	*Escherichia coli* GDH	IC_50_ > 100 μM	Li et al.^268^
Aurintricarboxylic acid (ATA)	Bovine GDH	IC_50_ 1.2 μM	Li et al.^257^
BH3I-2	Bovine GDH	IC_50_ 3.7 μM	Li et al.^257^
CK2 inhibitor	Bovine GDH	IC_50_ 15.8 μM	Li et al.^257^
BSB	Bovine GDH	IC_50_ 6.4 μM	Li et al.^257^
Calmidazolium	Bovine GDH	IC_50_ 7.7 μM	Li et al.^257^
Metergoline	Bovine GDH	IC_50_ 32.8 μM	Li et al.^257^
Sulocitidil	Bovine GDH	IC_50_ 13.8 μM	Li et al.^257^
Gallic acid	Bovine GDH	IC_50_ 80 μM	Li et al.^257^
Isophthalate (IP)	Bovine GDH	*K*i 300 μM	Aparicio et al.^269^
	*Clostridium symbiosum* GDH	*K*i 30 μM	Aparicio et al.^269^
	*Plasmodium falciparum* GDH	*K*i 6.2 μM	Aparicio et al.^269^
	*Aspergillus niger* NADP-GDH	IC_50_ ∼ 500 μM	Choudhury et al.^270^
2-methyleneglutarate	*Aspergillus niger* NADP-GDH	IC_50_ ∼ 500 μM	Choudhury et al.^270^

a The activities of these inhibitors were identified using GDH enzyme activity assays *in vitro*.

b The specific isoform of GDH could not be identified from the reference.

**Table 3:** Applications of GDH1/2 inhibition in cancers

Inhibitors	Type of cell lines	Species	Diseases	Models	Inhibition	Biological functions	Mechanisms	Reference
R162	H1299, MDA-MB231, A549, KG-1a, Molm14, K562, HEL, SKBR3	Human	LC BC Leukemia	*In vitro* *In vivo*	GDH1 activity	Inhibit proliferation	α-KG, GPx	Jin et al.^3^
	MOLM-13, MV4-11, OCI-AML2, OCI-AML3	Human	Leukemia	*In vitro* *In vivo*	GDH1 activity	Inhibit proliferation, combined toxicity with cytarabine	α-KG, GSH, GPx	Ma et al.^271^
	HCT116, C26	Human Mouse	CRC	*In vitro* *In vivo*	GDH1 activity	Induce apoptosis, especially used with carboxyamidotriazole	ROS, caspases	Shi et al.^272^
	A549/DTX	Human	LC	*In vitro* *In vivo*	GDH1 expression and activity	Inhibit proliferation and metastasis	ROS, Snail	Wang et al.^56^
EGCG	BCBL-1	Human	PEL	*In vitro*	GDH1 expression	Inhibit proliferation	α-KG	Yeh et al.^273^
	SF188	Human	GBM	*In vitro*	GDH1/2 activity	Induce cell death under glucose withdraw	α-KG	Yang et al.^51^
	A549/DTX	Human	LC	*In vitro* *In vivo*	GDH1 expression and activity	Inhibit proliferation and metastasis	Snail	Wang et al.^56^
	HCT116-IDH1^wt/R132H^	Human	CRC	*In vitro*	GDH1/2 activity	Inhibit proliferation, increase sensitivity	2-HG, γH2AX	Peeters et al.^274^
Decursinol angelate (DA)	HCT-116^MDR^	Human	CRC	*In vitro*	GDH1 expression and activity	Inhibit drug resistance	Rodex homeostasis, DNA repair, apoptosis	Chang and Kang^53^
Ebselen	A549 H22	Human Mouse	LC HCC	*In vitro*	GDH activity^a^	Inhibit proliferation	–	Hou et al.^260^
	Caki	Human	RCC	*In vitro*	GDH activity^a^	Inhibit proliferation	–	Ruan et al.^261^
Propylselen	A549 H22	Human Mouse	LC HCC	*In vitro*	GDH activity^a^	Inhibit proliferation	–	Hou et al.^260^
Hexylselen (CPD-3B)	A549 H22	Human Mouse	LC HCC	*In vitro* *In vivo*	GDH activity^a^	Inhibit proliferation	–	Fang et al.^275^
	A549, H22, HCT116, Caki-1, U251, SW1990, PC12	Human Mous	LC HCC CRC RCC GBM PDAC PCC	*In vitro* *In vivo*	GDH activity^a^	Inhibit proliferation	Necrosis, caspase-9, *p*-AKT, *p*-ERK1/2	Ruan et al.^261^
Canagliflozin	RPTEC/TERT1	Human	–	*In vitro*	GDH activity^a^	Induce cytotoxicity in proliferating cells	Anaplerosis	Secker et al.^263^
	BM-MSCs	Mouse	–	*In vitro* *In vivo*	GDH1 activity	Inhibit retention and paracrine function	ATP, cytochrome *c*, VEGFA secretion	Lin et al.^276^
Osmundacetone	A549, H460	Human	LC	*In vitro* *In vivo*	GDH1 expression	Inhibit proliferation	α-KG, NADH, ATP	Yang et al.^277^
Quercetin	HepG2	Human	HCC	*In vitro*	GDH1 activity	Inhibit proliferation	–	Marsico et al.^186^
Permethylated anigopreissin A (PAA)	HepG2	Human	HCC	*In vitro*	GDH1 activity	Inhibit proliferation	–	Marsico et al.^186^

Abbreviation in diseases: BC, breast cancer; CRC, colorectal cancer; GBM, glioblastoma; HCC, hepatocellular carcinoma; LC, lung cancer; RCC, renal cell carcinoma; PCC, pheochromocytoma; PDAC, pancreatic ductal adenocarcinoma.

a The specific isoform of GDH could not be identified from the reference.

### Purpurin and R162

Jin et al. identified purpurin as a lead compound for inhibiting GDH1 from a library of 2000 FDA-approved drugs.^3^ However, purpurin is not cell-permeable. In contrast, R162, a cell-permeable purpurin analog with an allyl group, exhibited more potent inhibitory effects on GDH1 activity in various cancer cell lines.^3^^,^^54^^,^^271^^,^^272^ In a thermal shift assay, incubating GDH1 with increasing concentrations of R162 led to a dose-dependent increase in the melting temperature (Tm), indicating direct binding of R162 to GDH1.^3^ In addition, in a competitive binding assay, where R162 was incubated with GDH1 in the presence of varying concentrations of α-KG, the Lineweaver-Burk plot revealed that R162 acted as a mixed-type inhibitor of GDH1. These results demonstrated that R162 directly targeted and inhibited the activity of GDH1.

Treatment with R162 results in decreased H1299 and MDA-MB-231 cell proliferation, while replenishing α-KG significantly rescued cell proliferation.^3^ R162 overcame DTX resistance in A549 cells by dual inhibition of GDH1 activity and expression, while also significantly reducing migration and invasion.^56^ The effect of R162 appears to be dependent on GDH1, as demonstrated by another study showing that the combination of R162 and FMK (an inhibitor of the pro-metastatic kinase RSK2) synergistically suppressed invasion and sensitized cancer cells to anoikis, an effect that was abolished in cells lacking GDH1 or RSK2.^55^

Interestingly, *GLUD1* knockdown or glutamine deprivation decreased cell proliferation in cancer cells, including H1299, MDA-MB-231, HEL, and K562, but had no effect on normal cells like MRC-5 and HaCaT, suggesting a crucial role for GDH1 in cancer metabolism.^3^^,^^31^^,^^50^ This phenomenon is also observed with R162 treatment,^3^ highlighting its antiproliferative potential in human cancer cells with minimal toxicity.

### EGCG and ECG

EGCG and its analog, epicatechin gallate (ECG), inhibited the activity of purified bovine GDH, whereas epigallocatechin (EGC) and epicatechin (EC) did not, although all four exhibited comparable antioxidant activities.^256^ The IC_50_ values for both EGCG and ECG are approximately 300 nM. It was also demonstrated that the inhibition of GDH by EGCG and ECG was independent of their antioxidant activity. EGCG inhibition depended on the antenna-like protrusion of bovine GDH, not the GTP binding sites, suggesting possible allosteric regulation rather than direct binding to the GTP site.^256^ However, studies conducted by Zhang et al. and Hou et al. challenged this view, demonstrating that EGCG also strongly inhibited *Escherichia coli* GDH, which lacks the antenna structure.^259^^,^^260^ Partial abrogation of EGCG inhibition by allosteric activators, such as ADP, leucine, or its nonmetabolizable analog 2-aminobicyclo-(2,2,1)-heptane-2-carboxylic acid (BCH), supported the notion that EGCG acted allosterically.^256^ Another study indicated that the binding sites of EGCG/ECG overlapped with the ADP allosteric binding sites of human GDH1, as demonstrated by structural analysis and site-directed mutagenesis, although with differences in their contact residues.^278^ Since the binding sites of EGCG and ECG were distant from the substrate binding sites, they were regarded as allosteric inhibitors.^256^^,^^257^^,^^278^^,^^279^

EGCG was also reported to modulate GDH1 expression. For instance, it downregulated GDH1 expression and induced cell death in primary effusion lymphoma cells.^273^ Furthermore, EGCG reduced GDH1 expression in DTX-resistant A549 cells and inhibited tumor growth *in vivo.*^56^ These studies indicate that EGCG inhibits both GDH1 activity and expression.

The toxicity of EGCG in effusion lymphoma cells was rescued by α-KG supplementation.^273^ EGCG-mediated inhibition of GDH1/2 enhanced sensitivity to low glucose and radiotherapy in CRC and GBM, which was also reversed by α-KG supplementation.^51^^,^^274^ Thus, while EGCG interacts with various cellular targets,^280^^,^^281^ inhibition of GDH1 is at least one of the key mechanisms.

### Decursin and decursinol angelate

Decursin (DN) and its isomer decursinol angelate (DA) are small natural compounds found in abundance in the roots of *Angelica gigas* Nakai, a medicinal herb in Asia, and exhibit various therapeutic properties, notably anti-cancer effects.^282^^,^^283^^,^^284^ DN and DA were selected via molecular docking analysis as GDH1 inhibitors from natural products, and their binding was verified in molecular dynamics simulation, demonstrating stable hydrogen bond interactions between DN or DA and the residues of R400 and Y386 at the ADP activation site of GDH1.^255^ Further biochemical analysis revealed that DN and DA inhibited GDH1 with IC_50_ values of 1.035 and 1.432 μM, respectively. Additionally, DA has been shown to dose-dependently inhibit GDH1 expression in multidrug-resistant HCT116 cells, leading to reduced α-KG levels and intrinsic apoptosis.^53^ It also synergistically enhanced the effect of *GLUD1* knockdown on cell proliferation while inhibiting ATP, fumarate, and α-KG production. However, whether the inhibition of DA on tumor progression is mediated through GDH1 has yet to be rigorously proven in these studies.

### Ebselen, propylselen, and hexylselen

Ebselen is a synthetic seleno-organic compound designed as a GPx mimic, with electrophilic and potential antioxidant properties.^285^ Its GPx-mimicking activity was well-characterized, in that the Se-N bond in ebselen is readily cleaved by thiols to form the corresponding selenol, which effectively reduces hydrogen/lipid peroxides.^286^ In addition, the thiol reactivity of ebselen enabled interactions with intracellular thiol-containing targets, including cysteine, GSH, and thiol proteins.^285^ It was reported that ebselen interacted with cysteine residues of chicken GDH, inducing the formation of an inactive hexameric form.^287^ Ebselen also interacted with *Saccharomyces cerevisiae* GDH3, inhibiting yeast growth in an ROS-dependent manner by increasing mitochondrial membrane potential, a mechanism similar to GDH3 deletion.^287^ Yu et al. reported that ebselen, along with its fluorescein- and biotin-labeled derivatives, inhibited *Escherichia coli* GDH activity by cross-linking to C321 at the catalytic site, adjacent to the NADP^+^ binding site and near the glutamate binding site.^262^ Due to the shared adjacent binding site, the interaction of ebselen derivatives with *E. coli* GDH could be competitively displaced by glutamate. It is worth noting that human GDH1/2 also contain several cysteine residues, suggesting a high likelihood that they are targets of ebselen. Hou et al. and Ruan et al. demonstrated the direct inhibition of human GDH (unspecified isoform) by ebselen.^260^^,^^261^

Despite its multiple pharmacological effects, including those on inflammation,^288^^,^^289^ oxidative stress,^290^^,^^291^ lung fibrosis,^292^ and neurodegenerative diseases,^293^^,^^294^ ebselen has also been shown to exhibit significant anti-tumor activity.^285^^,^^295^^,^^296^^,^^297^ For instance, ebselen induced cell-cycle arrest and caspase-independent cell death in LC cells through GSH depletion.^295^ Ebselen was also reported to trigger apoptotic pathways, resulting in cell death across various tumor types.^298^^,^^299^ However, due to the multitargeting nature of ebselen, its mechanism of action in cancer progression is complex and not exclusively attributed to GDH inhibition. Further investigations are needed to confirm whether GDH1/2 inhibition serves as the primary mechanism or functions alongside other pathways in mediating its antitumor activity.

Several other selenium-containing compounds have been shown to inhibit GDH activity. Propylselen, a dimeric derivative of ebselen, was a more potent human GDH (unspecified isoform) inhibitor targeting the NADP^+^ binding site and effectively suppressed liver and LC cell growth at lower concentrations than ebselen.^260^ Hexylselen (CPD-3B) has been identified as a dual inhibitor of KGA and GDH (unspecified isoform), exhibiting significantly stronger anticancer activity compared to ebselen.^261^^,^^275^ Hundreds of selenium-containing compounds have been synthesized,^300^ yet a systematic investigation into their inhibitory effects on GDH1/2 is lacking.

### Canagliflozin

Canagliflozin, a sodium-glucose co-transporter 2 (SGLT2) inhibitor, is currently utilized in clinical settings to treat hyperglycemia associated with type 2 diabetes. Recently, it has been found to exert inhibitory effects in pancreatic cancer and LC.^301^^,^^302^ Both Lin and Secker reported that canagliflozin was capable of inhibiting GDH1 activity, thereby hindering the TCA cycle and ATP production, leading to impaired cellular function.^276^^,^^263^ *In silico* docking analysis predicted that canagliflozin competitively bound to the ADP binding pocket of GDH1 and might abrogate the role of ADP in GDH1 activation.^276^ Further assays revealed restored GDH1 activity, ATP levels, and increased cell migration and proliferation in cells expressing mutant GDH1 (H199A and N392A), which were believed to be the key residues forming arene interactions within the GDH1 binding pocket that interacted with canagliflozin.

### Steroid hormones

Steroid hormones, including diethylstilbestrol (DES), estradiol, and progesterone, were reported to directly modulate bovine GDH activity.^265^ The inhibition of these steroids, as well as estriol, pregnenolone, corticosterone, dehydroepiandrosterone (DHEA), and dihydrotestosterone (DHT), was verified in human GDH1 and GDH2.^27^^,^^61^^,^^264^ The inhibition by steroids was significantly reversed by ADP, and no inhibition of either enzyme was observed in the presence of aldosterone. Among them, DES and corticosterone were the most potent inhibitors of GDH1/2, with IC_50_ values below 5 μM. Interestingly, corticosterone interacted with hGDH1 with a 2-fold greater affinity than with hGDH2, whereas other steroids exhibited at least 10-fold higher affinity for hGDH2 than for hGDH1.^27^ In human ovaries and placenta, both hGDH1 and hGDH2 were densely expressed in estrogen-producing cells, indicating that the selective affinity for estrogen may have important physiological significance. The application of steroid hormones in GDH inhibition within tumors has been sparsely reported.

### Others

Several natural products have been reported to inhibit GDH1. For instance, osmundacetone, a bioactive phenolic compound derived from *Phellinus igniarius*, downregulated *GLUD1* expression, disrupted the glutamine/glutamate/α-KG metabolic axis and OXPHOS, thereby inhibiting the proliferation of LC cells.^277^ Quercetin and permethylated anigopreissin A (PAA) dose-dependently inhibited bovine GDH activity, with IC_50_ values of 8 μM and 12 μM, respectively.^186^ The anti-tumor activity of quercetin and PAA has been reported in other studies, although the mechanisms have not been linked to GDH1 inhibition.^303^^,^^304^^,^^305^ Several chlorogenic acids were reported to inhibit bovine and chicken GDH without competing with glutamate or NAD^+^; however, their IC_50_ values were relatively high, ranging from 44 to 85 μM.^266^ Alpha-tocopherol inhibits both wild-type and H454Y mutant GDH1 *in vitro* and alleviates fasting hypoglycemia in a mouse model of HI/HA syndrome.^306^

Chlorinated compounds have also been identified as potential GDH1 inhibitors. Chloroquine has been shown to inhibit both human GDH1 and GDH2, with a higher inhibitory effect on GDH1 compared to GDH2.^267^ Nevertheless, the inhibition of GDH1/2 by chloroquine does not yet appear to be linked to its well-known pleiotropic effects, such as anti-malarial, anti-cancer, and anti-viral activities.^307^ Hexachlorophene (HCP) and bithionol (BTH) were identified as inhibitors against bovine GDH with IC_50_s in the low micromolar range using high-throughput methods to pan through more than 27,000 compounds.^257^ HCP binds as a ring within the innermost core of the hexamer, while BTH attaches to a different site located halfway between the core and the exterior of bovine GDH, distinct from HCP’s binding sites.^91^^,^^268^ Currently, there is no research reporting direct interactions between HCP or BTH and human GDH1/2, nor has any study linked their effects to the inhibition of GDH activity.

Several compounds, including aurintricarboxylic acid (ATA), BH3I-2, CK2 inhibitor GW5074, 3,3′-[(2-bromo-1,4-phenylene)di(E)ethene-2,1-diyl]bis(6-hydroxybenzoic acid) (BSB), calmidazolium, metergoline, sulocitidil, and gallic acid, have been identified as potential inhibitors of bovine GDH through high-throughput screening.^257^ However, their effects on human GDH1/2 have not been validated, and their role in oncology remains unclear.

Isophthalate (IP) inhibited both *Plasmodium* and bovine GDH, with a marked discrimination (70-fold lower Ki for the parasite GDH), thereby impairing the intra-erythrocytic *Plasmodium falciparum* growth.^269^ The dimethyl ester of isophthalate (DMIP) is readily absorbed and metabolized to IP inside cells. DMIP showed significantly higher inhibition of *Aspergillus niger* growth than IP, ascribed to the inability of IP to enter fungal mycelia.^270^ This also applied to 2-methyleneglutarate and its dimethyl ester, as reported for inhibiting *Aspergillus niger* NADP*-*GDH.

## Concluding remarks and future perspectives

**Figure 4:**
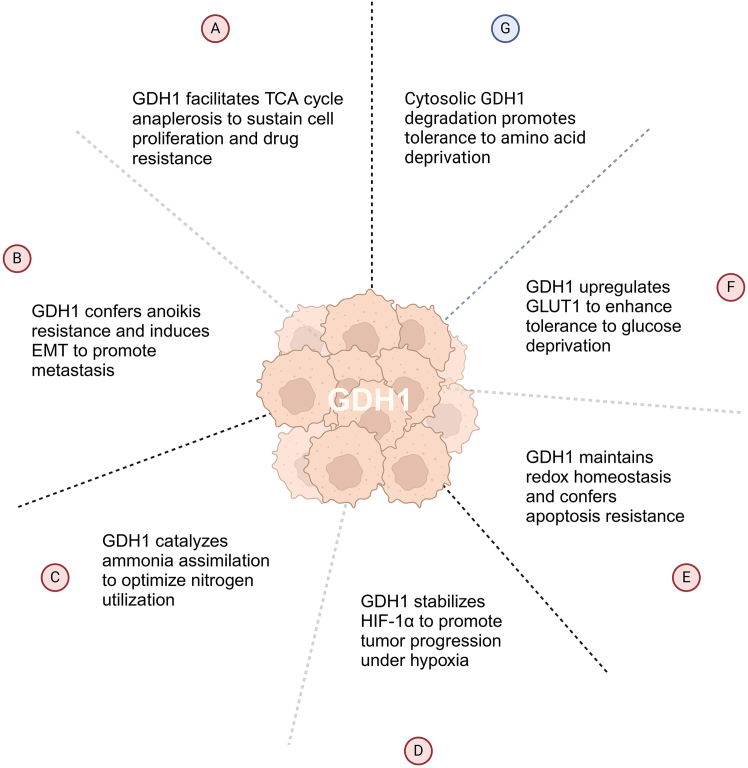
Multifaceted role of GDH in tumors (A) GDH1 facilitates TCA cycle anaplerosis to sustain cell proliferation and drug resistance. (B) GDH1 confers anoikis resistance and induces EMT to promote metastasis. (C) GDH1 catalyzes ammonia assimilation to optimize nitrogen utilization. (D) GDH1 stabilizes HIF-1α to promote tumor progression under hypoxia. (E) GDH1 maintains redox homeostasis and confers apoptosis resistance. (F) GDH1 upregulates GLUT1 to enhance tolerance to glucose deprivation. (G) Cytosolic GDH1 degradation promotes tolerance to amino acid deprivation. Created in BioRender. Sisi, Z. (2025) https://BioRender.com/n00r692.

GDH1 is widely expressed in both normal and cancerous tissues. Tumors are glutamine-dependent, utilizing two main pathways for its catabolism: GDH1- or transaminase-mediated glutaminolysis and the GTωA pathway. These pathways have nonredundant, tumor-specific functions. The role of GDH1 varies depending on the tumor’s origin, genetic background, metabolic environment, and disease stage. In most tumors, GDH1 facilitates α-KG production, generating energy and TCA cycle intermediates, many of which are considered oncometabolites and biosynthetic precursors. α-KG and oncometabolites (2-HG, succinate, and fumarate) competitively regulate 2-OGDDs, enzymes involved in epigenetic modifications (Figure 3). GDH1 also mediates the reductive amination of α-KG to maximize ammonia recycling under specific conditions. Additionally, GDH1 has been reported to modulate glucose transport, EMT, anoikis resistance, autophagy, HIF-1α stability, redox homeostasis, NF-κB signaling, and AMPK signaling in specific tumor types (Figure 4). GDH1 promotes tumor growth, metastasis, and survival under stress in most cases. However, GDH1 may function as a negative or irrelevant regulator in cancers such as KIRC, PDAC, and specific molecular subtypes of BC. Therefore, caution is warranted when considering GDH1 inhibition as a therapeutic strategy in these cancers. The enzymatic activity of GDH1 can be regulated by transcriptional, post-transcriptional, and allosteric mechanisms. Several GDH1 inhibitors have been reported; however, none have entered clinical trials, and the development of GDH1 inhibitors requires further investigation. Dual inhibition of GDH1 and GDH2 should be considered in the design of inhibitors targeting tumors with high GDH2 expression (especially in GBM) or tumors harboring IDH1/2 mutations. Furthermore, the impact of GDH1 inhibitors on normal cells, their potential side effects, and their combination with immune checkpoint inhibitors warrant further investigation.

### Limitations of the study

GDH1 has emerged as a key metabolic regulator in cancer. However, current evidence is limited to select cancer types, and its roles in other malignancies remain largely unknown. The diverse functions of GDH1, ranging from pro-tumorigenic to tumor-suppressive, further complicate its therapeutic targeting. To date, studies on GDH1 inhibitors have been confined to preclinical models, with limited clinical validation. Future research is needed to further characterize GDH1 across different cancers and elucidate its context-dependent mechanisms, providing insights for precision therapy.

## Acknowledgments

This work was supported by the 10.13039/501100003453Natural Science Foundation of Guangdong Province (2025A1515012020, 2021B1515020016) and the 10.13039/501100001809National Natural Science Foundation of China (81903914).

## Author contributions

S.Z.: Writing – original draft. H.W.: Writing – review and editing. Y.C.: Writing – original draft. J.L.: Writing – original draft. S.C.: Writing – original draft. H.Y.: Writing – review and editing. T.S.: Writing – review and editing. X.W.: Project administration, funding acquisition, writing – review and editing. L.X.: Project administration, funding acquisition, writing – review and editing.

## Declaration of interests

The authors declare no conflict of interest.
